# Maternal antibodies against the immunoglobulin M-degrading enzyme of *Streptococcus suis*, Ide_*Ssuis*_, play an important role in bactericidal immunity in young piglets in the field

**DOI:** 10.1186/s13567-026-01713-1

**Published:** 2026-03-19

**Authors:** Josepha Hallbauer, Jana Seele, Theresa Middendorf, Andreas Beineke, Peter Valentin-Weigand, Matthias Horn, Christoph G. Baums

**Affiliations:** 1 Institute of Bacteriology and Mycology, Centre for Infectious Diseases, Faculty of Veterinary Medicine, Leipzig University, An den Tierkliniken 29, 04103 Leipzig, Germany; 2 Institute for Microbiology, Centre for Infection Medicine, University of Veterinary Medicine Hannover, Bischofsholer Damm 15, 30173 Hannover, Germany; 3 Vivet Schweinegesundheit GmbH, Kahrweg 33, 59590 Geseke, Germany; 4 Department of Pathology, University of Veterinary Medicine Hannover, Bünteweg 17, 30559 Hannover, Germany; 5 Institute for Medical Informatics, Statistics and Epidemiology (IMISE), Faculty of Medicine, University of Leipzig, Härtelstraße 16-18, 04107 Leipzig, Germany

**Keywords:** IgG, colostrum, immune globulin protease, bactericidal assay

## Abstract

*Streptococcus* *suis* (*S.* *suis*) expresses a specific immunoglobulin M (IgM) protease, designated Ide_*Ssuis*_. Vaccination with recombinant Ide_*Ssuis*_ elicits protection against *S. suis* serotype 2. This study was initiated by the finding that the isogenic deletion mutant Δide_*Ssuis*_ shows a hypervirulent phenotype in 4-week-old piglets, presumably in association with maternally derived antibodies against Ide_*Ssuis*_. This prompted us to conduct a field study to test the working hypothesis that maternal αIde_*Ssuis*_ IgG antibodies are biologically relevant. Investigations in one herd with autogenous *S. suis* vaccination pre-farrowing showed a significant correlation of IgG levels against Ide_*Ssuis*_ in serum of 2-week-old-piglets and levels in colostrum. These antibody levels declined over the subsequent weeks of life. Levels of IgM binding to *S. suis* in sera of 2-week-old piglets did not correlate with respective levels in colostrum but with the amount of colostrum taken up. Bactericidal assays with wild type (wt) and ide_*Ssuis*_ mutants demonstrated that expression of functional or nonfunctional full-length Ide_*Ssuis*_ results in increased killing of *S. suis* serotype 2 in the blood of 2-week-old piglets. This phenotype disappeared as piglets aged. Linear mixed-effects models confirmed that serum IgG against Ide_*Ssuis*_ exerts a significant, time-independent effect on survival of *S. suis* wt and ∇ide_*Ssuis*__C195S, expressing a nonfunctional variant of Ide_*Ssuis*_, but not Δide_*Ssuis*_. Furthermore, IgM binding to *S. suis* wt influenced survival of wt and ∇ide_*Ssuis*__C195S in a time-dependent manner. This is in line with the concept that αIde_*Ssuis*_ IgG are protective through induction of fragment crystallizable (Fc)-mediated opsonophagocytosis rather than neutralization of the IgM protease, at least in suckling piglets.

**Supplementary Information:**

The online version contains supplementary material available at 10.1186/s13567-026-01713-1.

## Introduction

*Streptococcus suis* (*S. suis*) is a major pathogen in modern pig farming, causing septicemia, meningitis, and other pathologies. Worldwide, 29 serotypes have been identified. They are determined by capsular polysaccharides, which mediate protection against opsonophagocytosis [1, 2]. In addition, various proteins that interact with host factors are located on the surface of *S. suis*. Examples are proteins involved in transmigration through the blood–brain barrier such as muramidase-released protein (MRP) recruiting fibrinogen [3, 4], enolase binding plasminogen [5, 6], and factor H-binding protein (Fhb) [7].

The immunoglobulin M (IgM)-degrading enzyme of *Streptococcus suis,* Ide_*Ssuis*_, is also located on the streptococcal surface. This protease belongs to a family of highly specific immunoglobulin proteases. In contrast to the other members of this family, this cysteine protease cleaves only IgM and neither IgG nor IgA [8].

Ide_*Ssuis*_ is not crucial for virulence since isogenic mutants that do not express a functional IgM protease still cause disease to a high degree in experimental infections [9, 10]. However, in vitro experiments indicate that cleavage of IgM by Ide_*Ssuis*_ is a complement evasion mechanism used by *S. suis* [9, 10]. Various studies have confirmed that IgM plays an important role in the host defense against invasive *S. suis* infection [10–12]. Binding of IgM to *S. suis* serotype 2 in porcine blood leads to complement activation and oxidative burst of granulocytes, thereby mediating the killing of the streptococci [13]. After weaning, levels of IgM binding to *S. suis* increase prominently [11, 14]. IgM^+^CD21^−^ CD11R1^−^ B1-like cells play an important role as a source of IgM recognizing *S. suis* at around 8 weeks of age [15].

Colostrum is the only source of maternal IgG for neonatal piglets. Neonatal piglets should take up at least 250 g colostrum in the first 24 h of life [16, 17]. Levels of IgG decrease already in colostrum after 6 h after birth of the first piglet [18]. Porcine colostrum is known to also contain IgA and, to a very low degree, IgM [19, 20]. In the field, many dams are vaccinated with autogenous bacterins pre-farrowing, as a licensed vaccine eliciting protection against various serotypes is not available [21]. Vaccination of dams with *S. suis cps* 2 bacterins has been shown to elicit increased levels of IgG binding to streptococcal surface antigens in their piglets [22–24]. This might lead to protection against the homologous strain [24–26], but the specific targets of protective maternal IgG antibodies are not known.

IgG antibodies against the capsular polysaccharide of *S. suis* serotype (*cps*) 2 confer protection against this important serotype [25]. However, capsular polysaccharides are poor immunogens, and the role of these antibodies in maternal immunity is not well investigated. Vaccines that include recombinant Ide_*Ssuis*_ (rIde_*Ssuis*_) have been shown by independent research groups to elicit protection in pigs against different serotypes [26–28]. However, protection against a serotype 9 strain of sequence type (ST) 16 with a distinct ide_*Ssuis*_ allel, encoding a putative “group B IgM protease”, was not recorded. Sequence analysis revealed that 84–96% of confirmed clinical *S. suis* isolates carry the gene encoding the Ide_*Ssuis*_ protein, described as a protective antigen and designated type A IgM protease [26]. The “group B IgM protease” has not yet been characterized functionally. Still, antibodies elicited by vaccination do not cross-react between type A and type B antigen [26]. This study investigates the role of maternal IgG antibodies against type A Ide_*Ssuis*_, independent of vaccination with rIde_*Ssuis*_. We employed the bactericidal assay with different mutants to elucidate the roles of these antibodies and their putative mechanism for inducing bactericidal immunity in young piglets in a longitudinal field study.

## Materials and methods

### Bacterial strains and growth conditions

*S. suis* strain 10 is a virulent *cps* 2 strain of ST 1 expressing functional Ide_*Ssuis*_ on the bacterial surface [8]. It was used for generation of the isogenic deletion mutant 10Δide_*Ssuis*_ (Δide_*Ssuis*_), partial deletion mutants 10Δide_*Ssuis*__homologue and 10Δide_*Ssuis*__C_terminus, as well as a complemented mutant expressing Ide_*Ssuis*_ with a point mutation in the catalytic cysteine residue designated 10∆ide_*Ssuis*_∇ide_*Ssuis*__C195S (∇ide_*Ssuis*__C195S) [8, 10]. All these mutants of ide_*Ssuis*_ were generated in frame. The modified open reading frames are in control of the original ide_*Ssuis*_ promoter. The mutant Δide_*Ssuis*_ was also complemented previously with a plasmid carrying the entire ide_*Ssuis*_ gene and its promoter to generate strain Δ*ide*_*Ssuis*_ pGA14*ide*_*Ssuis*_ [8].

Streptococci were grown on Columbia agar plates with 5% sheep blood or in Bacto™ Todd Hewitt broth (THB). *Escherichia* *coli* (*E. coli*) BL21 carrying pET*ide*_*Ssuis*_ was cultured in Luria–Bertani medium with 100 μg/mL ampicillin [8].

### Expression and purification of recombinant Ide_*Ssuis*_ proteins

Recombinant His-tagged Ide_*Ssuis*_ (rIde_*Ssuis*_) was expressed in *E. coli* and purified after induction with isopropyl-β-d-thiogalactopyranoside using Ni^2+^-nitrilotriacetic acid affinity chromatography under native conditions, as described previously [8].

### Experimental infection of unvaccinated, 4-week-old piglets

The first experimental part of this study was conducted with German Landrace piglets originating from a specific pathogen-free (SPF) breeding operation, which was considered to be free of *sly*, *mrp*, *epf*, *cps* 2, and *cps* 9 strains, on the basis of the screening of more than 400 *S. suis* isolates collected from the tonsils of piglets over many years. Neither piglets nor dams were vaccinated against *S. suis* infection in the original herd.

The piglets were transported to the experimental facility 4 days after weaning. The experimental infection included 27 piglets, with 9 in each group infected with *S. suis* strain 10 (wt), Δide_*Ssuis,*_ or Δ*ide*_*Ssuis*_ pGA14*ide*_*Ssuis*_. The farm manager selected the litters and piglets from a litter (three or six) for this study without any specifications from the authors, except that they should be healthy, well-developed piglets. Each litter was represented by the same number of piglets in each group. Littermates were assigned to the different groups at random. This litter-matched design was conducted to minimize differences in genetics and former colostrum quality between the different infection groups.

At an age of 4 weeks, piglets were infected experimentally after predisposition through intranasal treatment with 3 mL of 1% acetic acid using an actuator (Valois Deutschland GmbH, Düsseldorf, Germany). This treatment and the experimental intranasal infection were conducted under anesthesia using an intramuscular (IM) injection of 2 mg/kg azaperone (Stresnil^®^, Janssen, Neuss, Germany) and 10 mg/kg ketamine hydrochloride (Ursotamin^®^, Serumwerk Bernburg, Germany). During the first anesthesia, blood was collected for serological and bactericidal assays. Streptococci were resuspended in phosphate-buffered saline (PBS) and applied intranasally 4 h after predisposition with the actuator, using an infectious dose of 2 × 10^9^ colony forming units (CFU; application of 1.5 mL with 1 × 10^9^ CFU to each nostril). The infectious dose was determined by serially diluting the inoculum and plating on Columbia blood agar plates within 2 h after experimental infection. Clinical monitoring was conducted every 8 h, including measurement of body temperature and assessment of feeding, behavior, and locomotion. Body temperature was measured rectally during feeding with a flexible clinical thermometer. Behavior and locomotion were assessed through observations before, during, and after feeding for at least 30 min at each monitoring time point using a sheet with predefined categories. In the case of high fever (≥ 40.5 °C), apathy and anorexia persisting over 36 h, as well as in all cases of clinical signs of acute polyarthritis or severe meningitis, animals were euthanized for reasons of animal welfare. All specific clinical signs, such as opisthotonus, convulsions, ataxia and recumbency to stand up, were confirmed by the animal experiment supervisor (CGB) prior to euthanasia. All surviving piglets were sacrificed 14 days post-infection. For euthanasia, piglets were first anesthetized with ketamine hydrochloride and azaperone. After confirmation of deep anesthesia, pentobarbital (80 mg/kg body weight) was injected intravenously.

### Pathohistological screenings of experimentally infected piglets

After euthanasia, every animal went through the same procedure of necropsy. The following samples were collected for histological (h) and bacteriological (b) investigations: cerebrospinal fluid (b); punctures of both tarsal and both carpal joints (b); tissues of both tarsal and both carpal joints (h); swabs of peritoneum, pleura, pericard, the mitral valve, and the brain (b); pleura and peritoneum (h); cranial lobe of the left lung (b, h); mitral valve and heart tissue, including pericard (h); spleen (b, h); tonsil (b, h); and brain (h). Fibrinous-suppurative inflammations were scored, as described previously, by a pathologist unaware of the clinical findings and the animal’s group affiliation [29]. To allow comparison of groups, the sum of the highest scores of each animal for any of the investigated organs was divided by the number of animals (*ω* = Σscore_max_/*n*_animals_). Isolation of the challenge strains was confirmed via polymerase chain reaction (PCR) for detection of *epf* and *cps* 2 [30] and in an *ide*_*Ssuis*_-specific PCR [9].

### Detection of αIde_*Ssuis*_ IgG and α*S. suis* IgM antibodies

Antibodies against Ide_*Ssuis*_ were measured in serum samples drawn form 4-week-old piglets of the experimental study, from 8-week-old piglets of a previously published experimental study [9], and in serum and colostrum samples collected in the barn during the longitudinal field study. Levels of α*S. suis* 10 IgM and αIde_*Ssuis*_ were measured using enzyme-linked immunosorbent assay (ELISA), as described [27], with the following modifications: MaxiSorp™ flat-bottom plates (Thermo Fisher Scientific, Darmstadt, Germany) were coated either with 0.2% formaldehyde-inactivated *S. suis* strain 10 or with 5 µg/well rIde_*Ssuis*_. Plates were blocked with 0.5% bovine serum albumin and 0.1% gelatin in PBS (pH 7.4) for 1 h at room temperature (RT). For detection of IgM and IgG, two goat peroxidase (POD)-labeled IgG antibodies were used, both with a dilution of 1:10 000: anti-pig-IgM (A100-117P, Bethyl Laboratories) and anti-pig-IgG (A100105P, Bethyl Laboratories). All samples and controls were measured in a duplicate series of four and the reference serum in a duplicate series of seven. Dilution series of sera and colostrum started with 1:100 (IgG-α-Ide_*Ssuis*_) or 1:50 (IgM-α-*S. suis* 10) and 1:3200 (IgG-α-Ide_*Ssuis*_) or 1:200 (IgM-α-*S. suis* 10), respectively.

For detection of α*S. suis* 10 IgM, serum of a bacterin-vaccinated piglet (no. 4515) was used as the reference serum and was defined as containing 100 ELISA units. Serum of a convalescent piglet (no. 238fbl) infected 3 weeks prior to the collection of this serum with *S. suis* strain 10 (wt) was used as a positive control. As in our previous vaccination studies [27, 28], serum of piglet no. 7376 immunized with rIde_*Ssuis*_ in a previous study [27] was defined as containing 100 ELISA units and served as the reference serum. Serum of piglet no. 9007 vaccinated with rIde_*Ssuis*__homologue served as positive control [27].

### Bactericidal assay

Survival of *S. suis* in porcine blood was determined as described [8]. In case of the experimental study, 500 µL of heparinized blood (16 IU/mL heparin) drawn from 4-week-old piglets (*n* = 6) was infected with 1.5 × 10^5^ CFU. These 6 piglets were randomly selected among the 27 piglets included in the experimental infection (sampling was conducted under anesthesia just prior to infection). These piglets originated from a German Landrace SPF breeding operation, which did not practice vaccination against *S.* *suis* infection. In the field study, heparinized blood (16 IU/mL heparin) was drawn from piglets (*n* = 72) in the barn at the indicated time points and transferred to the laboratory. Within 6 h after collection, 500 µL of blood was infected with 3 × 10^6^ CFU. In all cases, stocks of bacteria frozen with 15% glycerol were used after thawing. The blood was incubated for 2 h at 37 °C on a rotator. CFU were determined at *t* = 0 min (CFU_*t*0_) and *t* = 120 min (CFU_*t*120_) by serial dilution and plating on blood agar plates. The survival factor (SF) was calculated as CFU_*t*120_/CFU_*t*0_. For bactericidal assays with blood samples drawn during the field study, data transformation was performed (see the Statistical analysis section).

### Longitudinal field study in a herd practicing autogenous vaccination pre-farrowing

**Figure 1:**
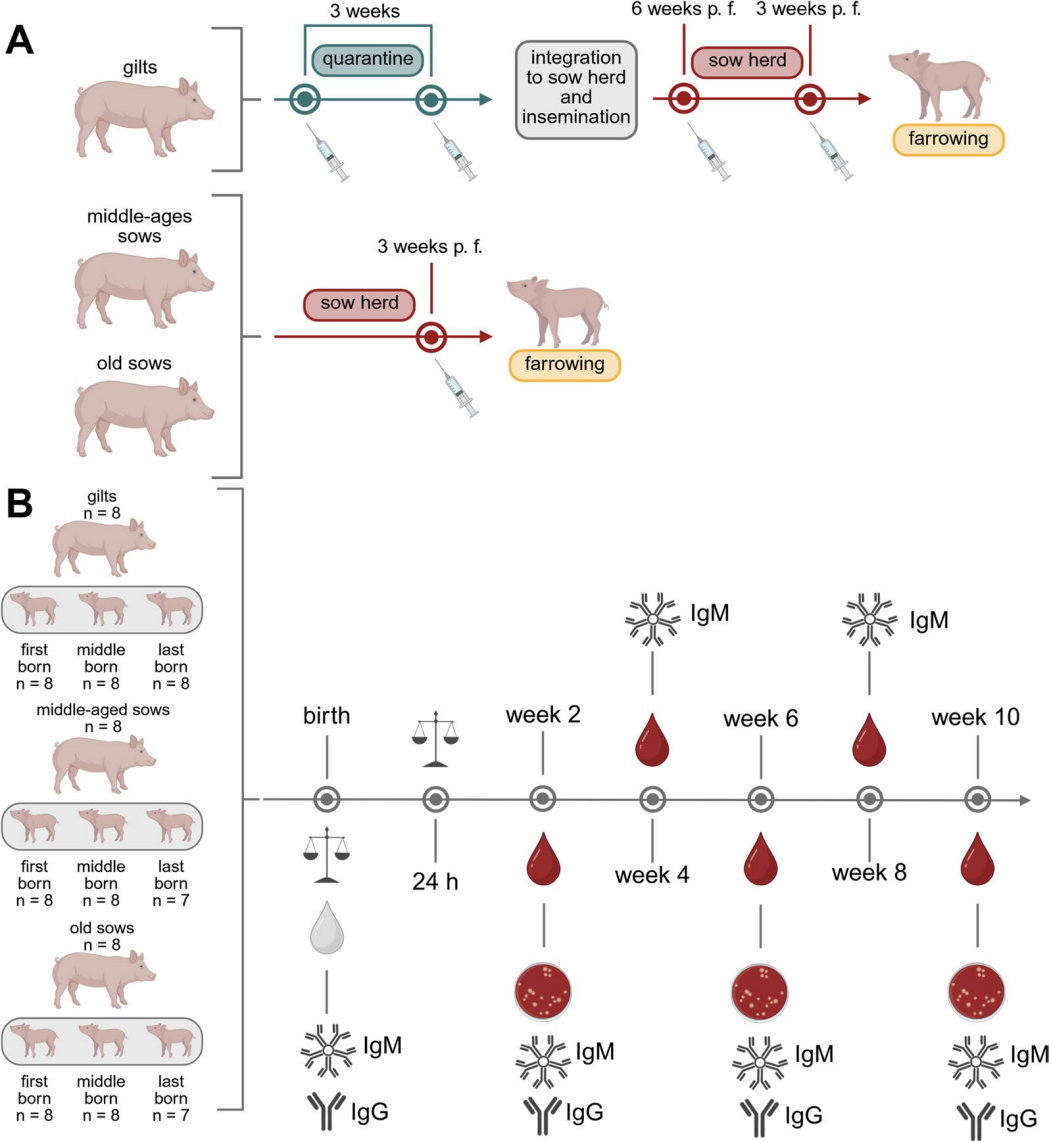
**Experimental design of the field study, including 24 dams and 70 of their piglets of a single herd**. **A** In this herd, dams are vaccinated with an autogenous bacterin, including, among other inactivated bacteria, *S. suis* *cps* 1 and *cps* 2 during quarantine of incoming gilts and pre-farrowing (p. f.) as indicated. **B** Sampling included collection of colostrum directly after the onset of birth of the first piglet and blood from the piglets at the indicated weeks of life. Readout parameters included determination of colostrum intake within the first 24 h using the formula by Theil et al. [31], measurement of levels of IgM and IgG binding to wt *S. suis* and rIde_*Ssuis*_, respectively, and determination of bacterial survival of *S. suis* wt, Δide_*Ssuis*_, and ∇ide_*Ssuis*__C195S. Data from the field study are shown in Figures 3, 4, 5, and 6 and Table 2.

A conventional pig farm with Hypor Libra × PIC408 genetics was selected for this field study. An autogenous multivalent bacterin with different bacteria, including *S.* *suis* *cps* 1 and *cps* 2 and 10% Emulsigen^®^ (oil-in-water emulsion, MVP Adjuvants, Phibro Animal Health Corporation, Teaneck, NJ, USA) as adjuvant was applied to incoming gilts two times during quarantine, as well as after integration and 6 and 3 weeks pre-farrowing. The *S. suis* *cps* 1 (no. 550) and *cps* 2 (no. 552) strains included in the autogenous vaccine were isolated from the joints of a diseased suckling piglet and weaning piglet in this herd in 2019 and 2016, respectively. Both strains are positive for the type A ide_*Ssuis*_ gene and the virulence-associated factors *mrp*, *sly*, and *epf*, and belong to ST 1. The sows were vaccinated again 3 weeks ante partum (Figure 1). The animal experiment included 24 dams (8 gilts, 8 middle-aged sows [second to fourth litter], 8 old sows [more than four litters]), and 70 of their piglets. The samples were collected during four different farrowing periods. In each farrowing period, two gilts, two middle-aged sows, and two older sows were included. The first born, middle-born, and last-born piglet of each dam was included in the study. In two litters, last-born piglets are not included in the analysis because one piglet was crushed by the sow in the first week of life (old sow) and one piglet of a middle-aged sow could not be identified in the weaning area (presumably owing to a lost ear tag). Suckling piglets of litters included in the experiment were not co-mingled. Serum samples of the piglets were taken from the cranial vena cava at an age of 2, 4, 6, 8, and 10 weeks of life. An additional heparinized blood sample was taken at 2, 6, and 10 weeks of life for bactericidal assays.

### Collection of colostrum samples and calculation of its intake (Cl_24_)

Immediately after the start of the birth of the first piglet, colostrum samples of three teats (first teat left, middle teat right, last teat left) were taken, cooled immediately, and stored at −80 °C until further use. To determine colostrum intake (CI) quantitatively, the piglets were weighted immediately after birth and exactly 24 h later. Colostrum intake in the first 24 h of life (CI_24_) was calculated using the formula described by Theil et al. [31]. In one piglet, data on birth weight and CI_24_ are missing.

### Statistical analysis

The data were presented as mean ± standard deviation (SD). Intergroup comparisons were executed through the implementation of conventional one-way or repeated measures analysis of variance (ANOVA) with Tukey’s multiple comparisons range test for post hoc analyses. In conventional linear regression analyses, a one-sample *t*-test was applied to the Pearson coefficients to assess the statistical significance of their deviation from zero (GraphPad Prism 10, USA).

A comparison of two independent groups for nonsymmetrically distributed data was performed using the Mann–Whitney *U* test. The differences in Kaplan–Meier diagrams were analyzed using the log-rank test. In this study, probabilities lower than 0.05 were considered as significant (^*^p < 0.05, ^**^p < 0.01, and ^***^p < 0.001).

The field study’s variables were transformed to obtain approximately normally distributed data. Levels of immunoglobulins measured with ELISA were logarithmized (log(1 + *x*)). The bacterial survival factors determined in bactericidal assays were Box–Cox transformed with *λ* = 0.13.

Linear mixed-effects models (LMMs) were employed to systematically investigate the impact of various parameters on the streptococcal survival factor at multiple time points. These parameters included CI_24_, the level of specific serum IgG against Ide_*Ssuis*_, and IgM binding to wt *S. suis*. This approach was employed to address the repeated measures design of the study and to adequately describe the dependency structure within the data, given that three piglets per dam were included.

LMM analyses were performed in R version 4.4.1 using the lme4 package [32]. For each bacterial strain that was investigated (wt, Δ ide_*Ssuis*_, ∇ide_*Ssuis*__C195S) we included survival factor as the dependent variable. To assess the effect of time (i.e., piglet age), we first specified a default model M1 as follows: M1: survival factor ~ time + (time | dam/piglet). Here we account for nested random effects of dams and piglets (denoted as dam/piglet) as well as for repeated measures (parameter time). Furthermore, the variable time was incorporated as a fixed effect, thereby enabling the extraction of the main effect of age, irrespective of the variability among individual dams and piglets. Subsequently, to evaluate the fixed effect of each parameter, two additional LMMs were fitted incrementally: M2: survival factor ~ time + parameter + (time | dam/piglet) and M3: survival factor ~ time * parameter + (time | dam/piglet). In M3, the asterisk represents a linear combination of both a fixed effect of the parameter (see M2) and a fixed interaction between piglet age (time) and the parameter (denoted as time:parameter). While in M2 the main effect was modeled to be consistent across all time points, M3 additionally evaluated the significance of an interaction between the age-related effect and the parameter effect.

For each parameter, a pairwise comparison of the models was performed using likelihood ratio tests. A significant difference between M2 and the default model M1 indicated that the parameter exerted an effect on the survival factor across the entire time period. Likewise, a significant difference between M3 and M2 suggested the presence of an interaction between the parameter and piglet age. The interaction was regarded as being inconsistent across the period if comparing M2 and M1 did not yield a significant improvement in model performance but comparison of M3 and M1 did.

The significance of the LMM coefficients was assessed by the lmerTest package [33], which employs Satterthwaite’s method to estimate degrees of freedom and generate *p*-values for LMMs. The 95% confidence intervals of the coefficient estimates were calculated using the bootstrap method with 10^5^ replicates.

## Results

### Three different ***ide***_***Ssuis***_ deletion mutants are less efficiently killed than the wt in porcine blood of 4-week-old, non-vaccinated piglets

**Figure 2:**
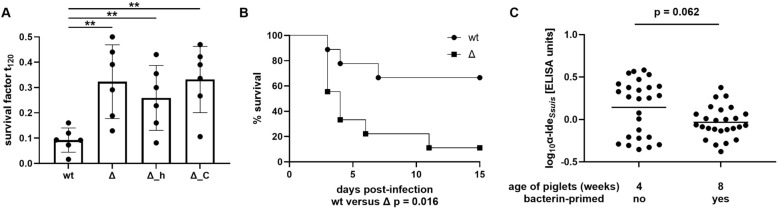
**Results of an experimental study demonstrating increased virulence of the deletion mutant 10Δ*ide***_***Ssuis***_ **in 4-week-old piglets**. **A** Survival of wild-type strain 10 (wt), Δide_*Ssuis*_ (Δ), 10Δ*ide*_*Ssuis*__homologue (Δ_h), and 10Δ*ide*_*Ssuis*__C-terminus (Δ_C) in porcine blood, which was drawn from six weaning piglets at an age of 4 weeks. **B** Kaplan–Meier survival diagram of unvaccinated weaning piglets (*n* = 9 per group) intranasally challenged with 2 × 10^9^ CFU of *S. suis* *cps* 2 strain 10 (wt) or the isogenic Δide_*Ssuis*_ (Δ) at an age of 4 weeks. **C** IgG levels against Ide_*Ssuis*_ in sera drawn from these piglets prior to challenge (**B**) in comparison with 8-week-old piglets of the same herd prime-vaccinated with a *S. suis* bacterin at 6 weeks of age within a different study [9]. The serum samples from the 8-week-old piglets were also collected prior to experimental infection. The data shown in this figure and in the previous study [9] were collected within the same year and independent of the data of the field study shown in the other figures. In **A** and **C**, bars and error bars represent mean values and standard deviations, respectively. Student’s* t*-test (**A**) or Mann–Whitney *U* test (**C**) was used for statistical analysis. Significant differences are indicated (^**^p < 0.01).

The IgM protease Ide_*Ssuis*_ mediates complement evasion and increased survival in porcine blood of 5–7-week-old piglets primed with a bacterin in association with high levels of specific IgM [10]. However, the level of IgM binding to *S. suis* also prominently increased in non-vaccinated piglets between 4 and 8 weeks of age [14, 15]. This study was initiated by the finding that three different ide_*Ssuis*_ mutants lacking different parts of the ide_*Ssuis*_ gene were, in comparison with the wt, less efficiently killed in blood drawn from 4-week-old piglets within an experimental study (Figure 2A). These piglets were from a herd (German Landrace SPF breeding operation) with a distinct *S. suis* infection status and no use of autogenous *S. suis* bacterins. This herd is known to be infected with a *cps* 2 strain of ST 28, which was also isolated from internal organs of single diseased animals in this herd [26] and carried the ide_*Ssuis*_ group A gene (results not shown). The bactericidal assays included, in addition to the complete mutant Δide_*Ssuis*_, the partial deletion mutants 10Δide_*Ssuis*__h and 10Δide_*Ssuis*_*_*C, lacking the IgM protease domain and the long C-terminal domain not related to IgM cleavage, respectively. In conclusion, expression of full-length Ide_*Ssuis*_ in *S. suis* wt results in increased killing in porcine blood of these 4-week-old weaning piglets originating from a *S. suis cps* 2-ST-28-infected herd.

### The deletion mutant 10Δ*ide*_*Ssuis*_ is hypervirulent in 4-week-old piglets

Four-week-old weaning piglets originating from this same breeding operation were infected intranasally with *S. suis* wt and Δide_*Ssuis*_ to access the virulence of both strains in piglets of this age. Infection with the mutant Δide_*Ssuis*_ caused 89% mortality, significantly higher than the mortality of 33% in the group infected with the wt (*p* = 0.016; Figure 2B). Accordingly, a high pathohistological score of 4.3, including four cases of severe meningitis, were recorded in the Δ*ide*_*Ssuis*_-infected group (Table 1; the score ranges from 0 to 5). In contrast, the pathohistological score of the wt-infected group was 2.8. Morbidity was 56% in wt-infected piglets and thus lower than the morbidity of 100% in the Δide_*Ssuis*_-infected piglets (*p* = 0.062).

**Table 1:** **Scoring of fibrinosuppurative lesions of weaning piglets after intranasal experimental infection with the indicated** ***S. suis*** **strains**

Infection strain	Piglets without lesions^a^	Piglets with lesions in three or more locations^a^	Brain				Serosae				Joint				Spleen and liver									Lung						Heart					Pathohistological score *ω*^f^
			Meningitis, chorioiditis				Pleuritis or peritonitis				Synovialitis				Splenitis^b^ or hepatitis							Pneumonia								Endocarditis					
			5^c^	3^d^	1^e^		4^c^	2^d^	1^e^		4^c^	2^d^	1^e^			4^c^	2^d^	1^e^					4^c^		2^d^	1^e^			4^c^		2^d^	1^e^			
wt (strain 10)	4/9	3/9	2/9	0/9	1/9		0/9	0/9	0/9		0/9	0/9	0/9			1/9	1/9	3/9					5/9		1/9	0/9			0/9		0/9	0/9			2.8
Δide_*Ssuis*_	2/9	7/9	5/9	0/9	0/9		1/9	0/9	0/9		4/9	1/9	0/9			0/9	3/9	2/9					7/9		2/9	0/9			0/9		0/9	0/9			4.3

^a^Only fibrinosuppurative lesions are considered.

^b^Neutrophilic accumulation of the splenic red pulp.

^c^Score of 4 and 5 indicates moderate-to-severe diffuse or multifocal fibrinosuppurative inflammation.

^d^Score of 2 and 3 indicates mild focal fibrinosuppurative inflammation.

^e^Individual single perivascular neutrophils received a score of 1.

^f^*ω* = Σscore_max_/*n*_animals_

This infection experiment also included a group infected with the complemented mutant Δ*ide*_*Ssuis*_ pGA14*ide*_*Ssuis*_, which showed a course of mortality similar to the mutant Δide_*Ssuis*_ and a pathohistological group score of 4.7 (results not shown). PCR screening of different isolates from inner organs of piglets infected with the complemented strain suggested that loss of the plasmid had occurred during the infection of these piglets in numerous cases (results not shown).

We have shown that vaccination of weaning piglets with rIde_*Ssuis*_ results in protection against *S. suis* strain 10 and increased killing of the wt in comparison with the Δide_*Ssuis*_ mutant [27]. We asked if the 4-week-old piglets used for the loss-of-function experiments carried increased levels of serum αIde_*Ssuis*_ IgG. Thus, we determined antibody levels against Ide_*Ssuis*_ in all sera drawn from piglets prior to experimental infection and from 8-week-old piglets originating from the same herd at the same time. The latter had been prime-vaccinated with a *S. suis* strain 10 bacterin at 6 weeks of age in an experimental study [9]. As shown in Figure 2C, the 4-week-old weaning piglets had higher levels of serum IgG against Ide_*Ssuis*_ than the older bacterin-primed, growing piglets, although the difference was not significant (*p* = 0.062). Differences in the αIde_*Ssuis*_ IgG levels between the differently infected groups were not observed (data not shown). In conclusion, loss of Ide_*Ssuis*_ expression leads to hypervirulence in 4-week-old piglets in association with increased survival in blood and increased αIde_*Ssuis*_ serum IgG levels.

### Detection of maternal IgG against Ide_*Ssuis*_ in 2-week-old suckling piglets in a herd practicing autogenous bacterin vaccination pre-farrowing

**Figure 3:**
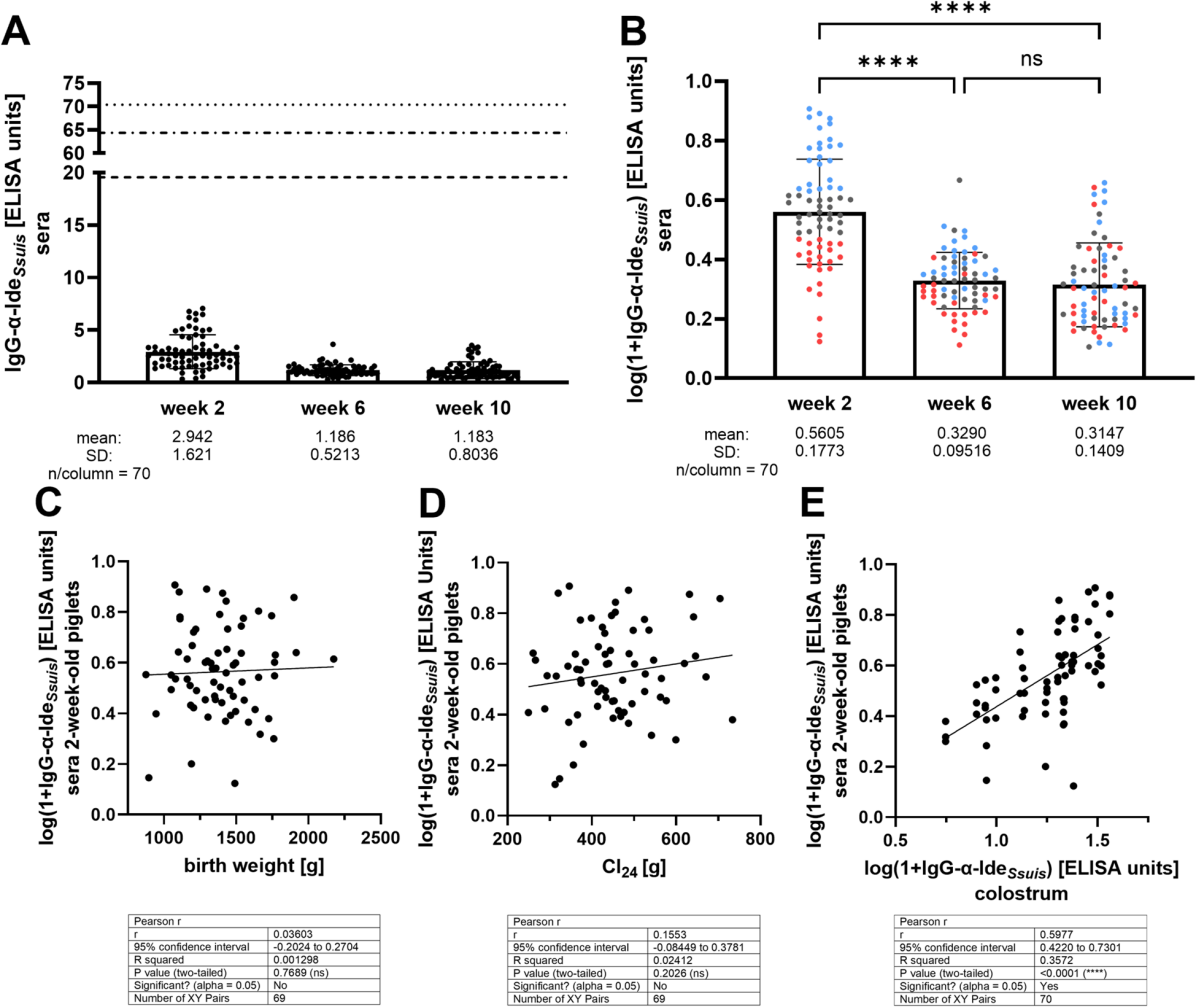
**IgG antibody levels against Ide**_***Ssuis***_ **in piglets and colostrum in a herd practicing autogenous bacterin vaccination pre**-**farrowing**. **A** Serum IgG levels against Ide_*Ssuis*_ in 2-, 6-, and 10-week-old piglets in ELISA units in comparison with levels observed in previous studies after vaccination with rIde_*Ssuis*_: Seele et al. (dotted line) [27], Rieckmann et al. (dotted–dashed line)[28], and Mayer et al. 2024 (dashed line) [34]. **B** Logarithm of values shown in (**A**) and coloring according to levels at 2 weeks of age. **C–E** Correlation analysis between specific serum IgG αIde_*Ssuis*_ levels and birth weight (**C**), colostrum intake in the first 24 h (CI_24_) determined as described previously [31] (**D**), and the levels of αIde_*Ssuis*_ IgG in colostrum (**E**).

**Figure 4:**
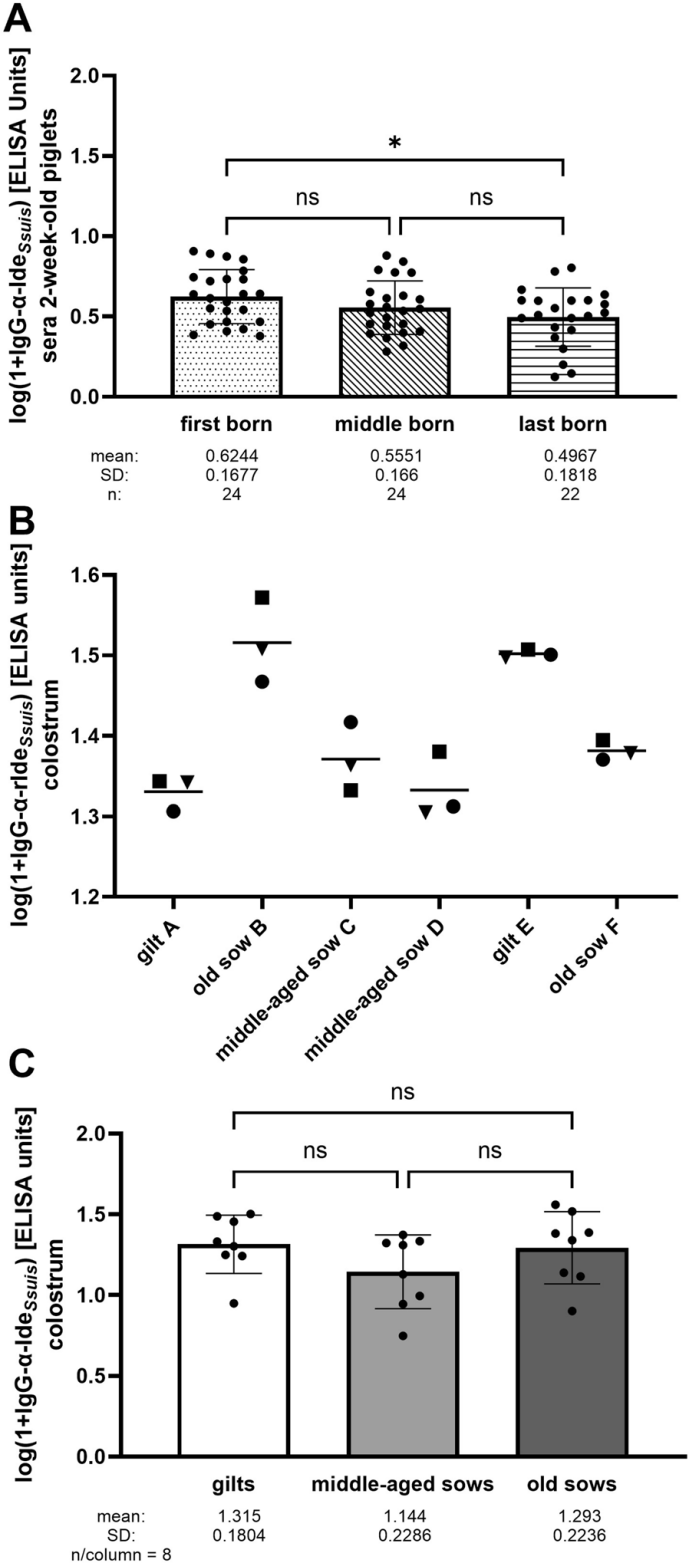
**Comparison of IgG αIde**_***Ssuis***_ **levels in sera drawn from 2-week-old piglets in dependence of farrowing order **(**A**) **as well as in colostrum samples drawn from different teats of the indicated dams** (**B**)** and from different groups of dams** (**C**). **A** Sera of 2-week-old first-, middle-, and last-born piglets of the longitudinal field study were collected as indicated in Figure 1. Each litter (*n* = 24) is represented by one animal in each of the indicated groups with the exception of two missing last-born piglets. **B** Three teats of each dam were sampled to obtain colostrum. The indicated dams belonged to the group that farrowed in the first period used for sampling. IgG αIde_*Ssuis*_ levels were determined in colostrum samples of different teats. **C** Comparison of αIde_*Ssuis*_ IgG levels in pooled colostrum samples of gilts, middle-aged sows, and old sows from the entire study (each *n* = 8 and four sampling periods).

The data of the experimental study shown in Figure 2 prompted us to investigate the working hypothesis that piglets in the field carry maternally derived IgG against Ide_*Ssuis*_, which might be important for bactericidal immunity. To investigate the transfer of maternal IgG against Ide_*Ssuis*_ in the field, we selected a herd with autogenous *S. suis* bacterin vaccination pre-farrowing. Sera were collected from 70 different piglets at 2, 6 and 10 weeks of age. Levels of IgG against Ide_*Ssuis*_ were below 8 ELISA units in all investigated samples (Figure 3A) and thus much lower in comparison with levels recorded after prime-boost vaccination with rIde_*Ssuis*_ in 8-week-old growing piglets in our previous studies [27, 28, 34]. For further analysis, values were first logarithmized to obtain an approximately normal distribution (Figure 3B). IgG against Ide_*Ssuis*_ obtained a mean of 0.5605 (± 0.1773) (1 + log αIde_*Ssuis*_) and showed a highly significant decline between 2 and 6 weeks of age. Though the mean of αIde_*Ssuis*_ IgG remained at the same level between 6 and 10 weeks of age, single piglets showed an increase between the 6^th^ and 10^th^ week of life (Figure 3B). These single piglets were found in the different thirds defined by the αIde_*Ssuis*_ IgG levels at 2 weeks of age (see different colors in Figure 3B). At 2 weeks of age, levels of serum αIde_*Ssuis*_ IgG were significantly increased in first-born in comparison with last-born piglets (*p* = 0.0373) (Figure 4A).

Measurement of αIde_*Ssuis*_ IgG levels in colostrum samples drawn from different teats in the first sampling period revealed that the levels were very much the same between teats of the same dam (Figure 4B). Accordingly, samples from three different teats of one dam were pooled for further analysis. No significant differences were observed between αIde_*Ssuis*_ IgG levels in colostrum drawn from gilts, middle-aged sows, and old sows with mean (1 + log αIde_*Ssuis*_) IgG values of 1.315 (± 0.1804), 1.144 (± 0.2286), and 1.293 (± 0.2236), respectively (Figure 4C). Correlation analysis revealed that αIde_*Ssuis*_ IgG levels at 2 weeks of age did not correlate with birth weight (Figure 3C) or colostrum intake in the first 24 h (Figure 3D) but with the level of αIde_*Ssuis*_ IgG in colostrum (Figure 3E). The latter finding, along with the observed decline between 2 and 6 weeks of age, confirms that maternal αIde_*Ssuis*_ IgG are detectable in young piglets in this herd.

### Levels of IgM recognizing *S. suis* *cps * 2 are very low in 2-week-old piglets suckled by bacterin-vaccinated dams and do not correlate with respective levels in colostrum

**Figure 5:**
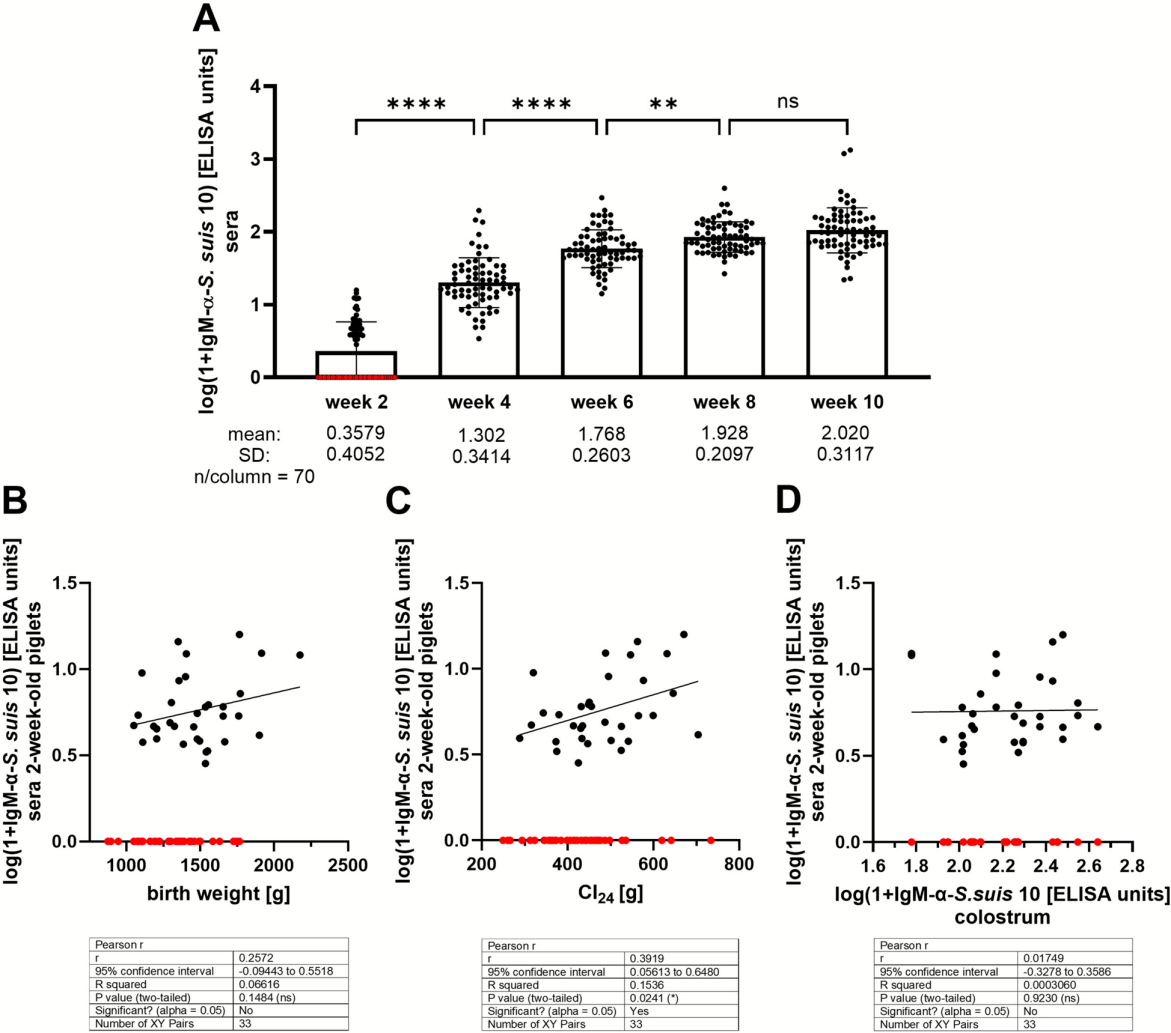
**Levels of serum IgM binding to  *S. suis cps *2** **(specific IgM) in piglets of dams vaccinated with an autogenous bacterin as depicted in Figure** 1. **A** Course of specific IgM in sera of piglets (*n* = 70) sampled every 2 weeks after the 2^nd^ week of life. **B–D** Correlation analysis of specific IgM levels in sera of 2-week-old piglets with birth weight (**B**), colostrum intake in the first 24 h (CI_24_) (**C**), and specific IgM levels in colostrum samples of the respective dams (**D**). Points marked in red on the *x*-axis refer to samples of 2-week-old piglets that obtained values below the detection level of our IgM ELISA. These cases are not included in the correlation analysis shown in **B**–**D.**

Levels of IgM antibodies binding to *S. suis* increase prominently between the 2^nd^/4^th^ and 8^th^ week of life. This has been shown by our group for different serotypes and piglets from different herds [11, 14, 15]. An increase of IgM binding to *S. suis* *cps* 2 between 2 and 8 weeks of age was also confirmed for the piglets investigated in this field study (Figure 5A). At 2 weeks of age, these levels were very low. In 53% of the investigated 2-week-old piglets (*n* = 70), the level of IgM binding to *S. suis* wt was below the detection level of our ELISA (red dots in Figure 5A). Nevertheless, we investigated if the positive levels in the remaining samples correlated with birth weight, colostrum intake, and respective levels in colostrum. The levels of IgM binding to *S. suis* wt did not correlate with birth weight (Figure 5B). In the case of positive samples, levels of IgM binding to *S. suis* *cps* 2 showed a significant correlation with colostrum intake (Figure 5C) but no correlation with respective IgM levels in colostrum (Figure 5D). Overall, it is very questionable that sera of 2-week-old piglets contain maternal IgM recognizing *S. suis* in this herd.

### Expression of Ide_*Ssuis*_ or nonfunctional Ide_*Ssuis*__C195S is associated with significantly increased killing of *S. suis* *cps * 2 in blood of 2-week-old piglets

**Figure 6:**
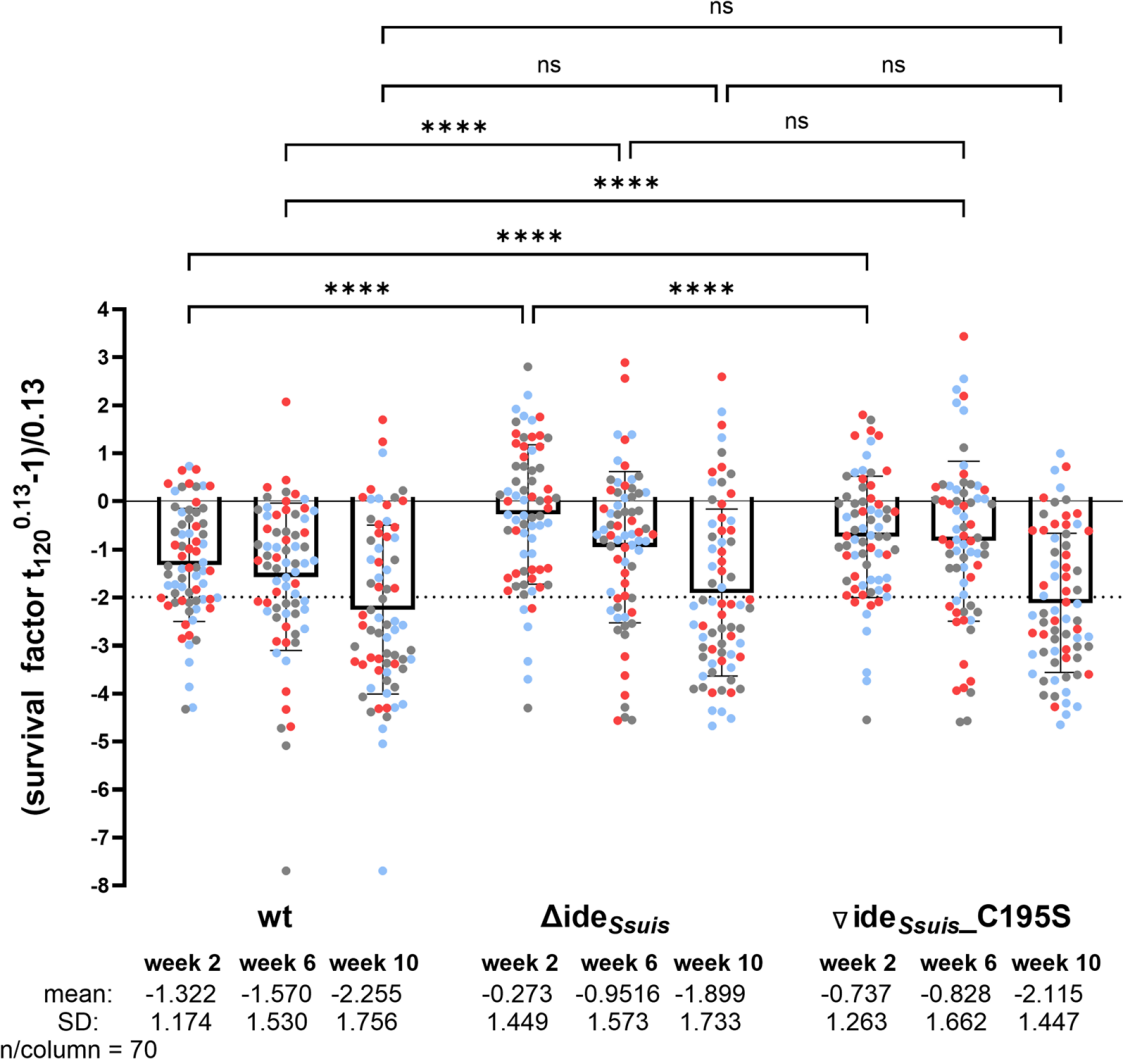
**Comparison of survival of** ***S. suis*** **wt** **(strain 10), its isogenic ide*Ssuis*** **deletion mutant** **Δ** **ide**_***Ssuis***_**, and a mutant expressing a nonfunctional, full-length point-mutated variant of Ide**_***Ssuis***_ **(∇ide**_***Ssuis***_**_C195S).** The three colors refer to the level of IgG antibodies against Ide_*Ssuis*_ at 2 weeks of age, as shown in Figure 3B, with blue and red indicating relatively high and low levels, respectively. Bacterial survival factors (SF) determined in bactericidal assays were Box–Cox transformed with *λ* = 0.13 to obtain approximately normally distributed values. A value of 0 is obtained for an original SF of 1. An SF above 0 indicates proliferation, and an SF below 0 indicates killing of the bacteria. The dotted line (SF = −1.99) indicates killing of 90% of the starting inoculum and is equivalent to an original SF of 0.1. Blood was drawn from the same animals at all time points.

In our rIde_*Ssuis*_ vaccination trial with weaning piglets, bactericidal assays revealed efficient killing of *S. suis* wt in blood of vaccinated piglets in contrast to non-vaccinated littermates [27]. The isogenic mutant Δide_*Ssuis*_ showed survival in blood samples of rIde_*Ssuis*_-vaccinated piglets [27]. Here, we conducted bactericidal assays with *S. suis* wt, Δide_*Ssuis*_, and ∇ide_*Ssuis*__C195S with all blood samples collected from the 70 piglets at 2, 6, and 10 weeks of age. We observed significantly increased survival of the isogenic mutant Δide_*Ssuis*_ compared with the other two strains in blood samples of 2-week-old piglets (Figure 6). Specifically, this mutant proliferated in blood of 33 of the 70 investigated piglets, whereas this was true for the wt and ∇ide_*Ssuis*__C195S in 10 and 17 piglets, respectively. Indeed, more than 80% of the *S. suis* wt bacteria were killed in the blood of 32 of 70 2-week-old piglets (Δide_*Ssuis:*_ only in 16 piglets). In the following 4 or 8 weeks of life, the mean survival factors of all three investigated strains decreased, resulting in significantly increased killing in 10-week-old piglets of all three strains (Figure 6). At this age, the majority of piglets showed efficient killing of all three strains, though there were still substantial differences in killing efficiency between the different piglets. Significant differences in survival of wt and the two mutants were not recorded in blood samples drawn at 10 weeks of age.

### Levels of αIde_*Ssuis*_ IgG show a significant, time-independent effect on survival of *S. suis *wt in blood of 2-, 6-, and 10-week-old piglets on LMM analysis

We used linear mixed-effects models to explore the effects of different variables on killing of *S. suis* wt, Δide_*Ssuis*_, and ∇ide_*Ssuis*__C195S in blood of 2-, 6-, and 10-week-old piglets. Litter order, parity of the dam, birth weight of the piglet, colostrum intake, and body weight at weeks 2, 6, and 10 post-partum showed no influence on streptococcal survival factors (Additional file 1). In contrast, levels of specific IgG against Ide_*Ssuis*_ in sera of piglets had a time-independent, highly significant (*p* < 0.001) effect on survival of *S. suis* wt and ∇ide_*Ssuis*__C195S but no effect on survival of Δide_*Ssuis*_ (Table 2). On the basis of the interaction model (M3), levels of IgM binding to *S. suis* wt showed a time-dependent effect on survival of *S. suis* wt and ∇ide_*Ssuis*__C195S.

**Table 2:** **Selected results of linear mixed-effects model M2 on survival of different** ***S.*** ***suis*** ***cps*** **2 bacteria strains in blood of 2-, 6-, and 10-week-old piglets**

Bacterial strain	Parameter	Estimate β	95% confidence interval	SE	*p*-Value
wt	Time	−0.1860	[−0.2885, −0.0833]	0.0520	0.0012
	αIde_*Ssuis*_ IgG	−0.9723	[−1.5410, −0.4060]	0.2821	< 0.001
Δide_*Ssuis*_	time	−0.2282	[−0.3350, −0.1213]	0.0541	< 0.001
	αIde_*Ssuis*_ IgG	−0.3792	[−1.0464, 0.2899]	0.3305	0.2528
∇ide_*Ssuis*__C195S	time	−0.2418	[−0.3375, −0.1453]	0.0488	< 0.001
	αIde_*Ssuis*_ IgG	−0.9572	[−1.5942, −0.3239]	0.3166	0.0029

In conclusion, expression of functional or nonfunctional, full-length Ide_*Ssuis*_ in *S. suis* *cps* 2 results in increased killing of streptococci in blood of 2-week-old suckling piglets of this herd in association with the presence of maternal αIde_*Ssuis*_ IgG. Levels of αIde_*Ssuis*_ IgG and IgM binding to *S. suis* *cps* 2 play a time-independent and time-dependent role in killing of wt *S. suis* in blood of piglets within the critical phase of piglet rearing, respectively.

## Discussion

IgM plays an important role in control of invasive *S. suis* infection and killing of the bacteria in blood, in particular [12, 13, 35]. The binding of IgM to the streptococcal surface is an important mechanism to mediate opsonophagocytosis in porcine blood via complement activation [13]. In this field study, we showed that at 2 weeks of age, the investigated piglets had very low levels of serum IgM binding to the streptococcal surface (Figure 5A). A correlation between levels of specific IgM in sera of 2-week-old piglets and colostrum of their dams was not recorded (Figure 5D). This suggests that maternal IgM plays most likely only a minor role in protection against invasive *S. suis* infection at this age. Already between the 2^nd^ and 4^th^ week of life, we observed a highly significant increase in levels of serum IgM binding to *S. suis* (Figure 5A). These levels increased further until an age of 8 weeks, in agreement with previous studies [14, 15]. We speculate that the amount of colostrum taken up has a positive effect on the early generation of IgM recognizing *S. suis* and that some piglets already carry self-developed IgM recognizing *S. suis* at an age of 2 weeks. This would explain the positive correlation of levels of serum IgM recognizing *S. suis* in 2-week-old piglets with colostrum intake but not with respective levels of colostrum (Figures  5 C and D).

LMM analysis revealed that levels of specific IgM exhibit a time-dependent effect on survival of *S. suis* wt (and ∇ide_*Ssuis*__C195S but not Δide_*Ssuis*_) in porcine blood. In agreement, our group observed a significant increase of the survival factors of *S. suis* *cps* 1 and *cps* 14 in blood of 6- and 8-week-old but not 2-week-old piglets of a different herd upon cleavage of IgM through addition of rIde_*Ssuis*_ [14]. The preferred interaction model (M3) indicates that, as time increases, a lower level of IgM binding to *S. suis* wt is sufficient for the same effect size, i.e., reduction of the survival factor. However, our findings demonstrate that IgM recognizing *S. suis* wt increases over time. Consequently, the decline in the survival factor becomes more pronounced as time increases. A possible explanation for the preference for M3 is that as maternal, IgG-dependent immunity decreases during piglet rearing, the effect size of IgM mediating killing of *S. suis* gets greater. However, it was surprising that LMM analysis did not reveal a significant effect of IgM levels on survival of Δide_*Ssuis*_. As determined by injection of iodine-125-labeled antibodies, the mean half-lives of plasma IgM and IgG in neonatal piglets are 2.8 and 9.1 days, respectively [36]. Thus, it is a limitation of this study that the first blood sample was not taken in the first days of life to assess transfer of maternal IgM. It appears likely that maternal IgM declined rapidly within the first 2 weeks of life, leading to the very low levels at week 2.

In this study, we demonstrate in two independent assays thatthe isogenic deletion mutant Δide_*Ssuis*_ is less efficiently killed in blood of 4- or 2-week-old piglets in comparison with the wt. All piglets originated or belonged to herds infected with *S. suis* strains expressing type A IgM protease. The increased survival of the isogenic Δide_*Ssuis*_ mutant in blood is in accordance with hypervirulence of this mutant in experimental infection of young piglets. Hypervirulence of mutants has been observed for regulators of virulence factors such as the CovR/S two-component system in *Streptococcus pyogenes* [37] but also for serine protease autotransporters of *Enterobacteriaceae* (SPATEs). Specifically, the SPATE null mutants Δcrc1 and ΔpicC of *Citrobacter rodentium* cause hyperinflammation, the latter owing to increased Toll-like receptor 2 (TLR2) recognition [38, 39]. However, it appears very unlikely that loss of function of Ide_*Ssuis*_, other than recognition by antibodies, is involved in the hypervirulent phenotype. Complementation of the mutant with a point mutant lacking IgM protease activity results in a significant increase in killing of *S. suis* in porcine blood (Figure 6). One might argue that the large C-terminal domain of Ide_*Ssuis*_ is not necessary for IgM cleavage and might carry out an additional, not yet discovered, function, reducing virulence in the wt. However, as shown in Figure 2, deletion of the N-terminal IgM protease domain alone in 10Δ*ide*_*Ssuis*_ _homologue is sufficient to generate a variant with increased survival in porcine blood of young piglets. This mutant is expected to express the C-terminal part of Ide_*Ssuis*_ in association with the bacterial surface similar to wt *S. suis*. Most importantly, linear mixed-effects models revealed that serum αIde_*Ssuis*_ IgG levels have an influence on survival of *S. suis* and ∇ide_*Ssuis*__C195S but not Δide_*Ssuis.*_

The findings that the complemented mutant ∇ide_*Ssuis*__C195S is killed more efficiently than Δide_*Ssuis*_ in blood of 2-week-old piglets (Figure 6) and that all three independent deletion mutants show comparable phenotypes in the bactericidal assay (Figure 2A), exclude the alternative explanation that a spontaneous mutation in the chromosome not related to ide_*Ssuis*_ is related to the reduced killing in blood. We conclude that the hypervirulent phenotype of the mutant Δide_*Ssuis*_ in bactericidal assays and experimental infection of young piglets is caused by serum αIde_*Ssuis*_ IgG mediating killing of *S. suis* expressing full-length Ide_*Ssuis*_. This is in accordance with the high protective efficacy of rIde_*Ssuis*_ vaccination against *S. suis* *cps* 2 of ST 1 [26, 27].

The decline of levels of αIde_*Ssuis*_ IgG in piglets between 2 and 6 weeks of age (Figures 3A and B) as well as the high correlation of levels of serum αIde_*Ssuis*_ IgG in 2-week-old piglets and matched colostrum samples justify the conclusion that serum αIde_*Ssuis*_ IgG antibodies of 2-week-old piglets are mainly of maternal origin in this herd. Jacobs et al. [26] recorded protection against experimental *S. suis* *cps* 2 infection in 8-week-old piglets after pre-parturient sow vaccination with rIde_*Ssuis.*_ This long-lasting, protective maternal immunity after rIde_*Ssuis*_ vaccination pre-farrowing is in agreement with the conclusion that even comparatively low levels of maternal αIde_*Ssuis*_ IgG are biologically very relevant.

It was not possible to include a group of unvaccinated dams in the longitudinal field study for reasons related to regulations of animal experiments in Germany. Accordingly, the field study does not allow for an evaluation of whether application of the autogenous vaccine elicited αIde_*Ssuis*_ IgG in dams. However, the results shown in Figure 2 were obtained with piglets originating from a different herd where no autogenous *S. suis* vaccine is used. As the phenotype of the Δide_*Ssuis*_ mutant in the respective bactericidal assay (Figure 2A) is in accordance with the results of the field study (Figure 5), this study suggests that biologically relevant levels of maternal αIde_*Ssuis*_ IgG are also present in unvaccinated piglets in the field.

There are different possible explanations for the protection elicited by rIde_*Ssuis*_ vaccination. As Ide_*Ssuis*_ also cleaves the IgM B cell receptor (BCR) and leads to modulation of B cell signalling [40, 41], one might speculate that αIde_*Ssuis*_ antibodies prevent binding and processing of the BCR by Ide_*Ssuis*_. This hypothesis is, however, currently speculative, because a different immune response to *S. suis* infection in vivo due to Ide_*Ssuis*_ activity has not been demonstrated. Furthermore, it does not explain the results of the bactericidal assay in rIde_*Ssuis*_-vaccinated animals showing efficient killing of the wt but not the isogenic mutant Δide_*Ssuis*_ [27]. Another possible explanation is that inhibition of cleavage of IgM binding to the streptococcal surface by αIde_*Ssuis*_ antibodies is crucial for protection. Of note, we have demonstrated in previous studies that antibodies elicited by rIde_*Ssuis*_ vaccination might neutralize the IgM protease activity [27, 34]. Activation of the complement-oxidative burst axis by IgM is an important defensive mechanism against *S. suis* bacteremia [13]. Accordingly, increased activation of complement on the streptococcal surface would be expected in piglets with increased levels of αIde_*Ssuis*_ IgG and IgM binding to the streptococci. However, 2-week-old piglets with detectable maternal αIde_*Ssuis*_ IgG levels showed very little serum IgM binding to *S. suis* (Figure 4). Of note, these levels were below the detection level of our ELISA in numerous piglets. In accordance, IgM was found to exert a time-dependent effect on survival of wt *S. suis* in the bactericidal assays in LMM analysis, implying that survival of *S. suis* is limited by IgM in blood of 6- and 10-week-old but not 2-week-old piglets. On the basis of these findings, it appears very unlikely that neutralization of the IgM protease activity is involved in the ide_*Ssuis*_-dependent phenotypes in bactericidal assays run with blood of 2-week-old piglets (Figure 6). Finally, αIde_*Ssuis*_ IgG might directly opsonize *S. suis* *cps* 2 and activate Fc_ϒ_-mediated phagocytosis. The results of the bactericidal assay shown in Figure 6 are in accordance with this hypothesis, because there is a highly significant phenotypic difference between Δide_*Ssuis*_ and ∇ide_*Ssuis*__C195S in survival in blood of 2-week-old piglets that cannot be explained by neutralization of the IgM protease by maternal αIde_*Ssuis*_ IgG, because both strains do not cleave porcine IgM [10].

The epitopes of Ide_*Ssuis*_ recognized by protective αIde_*Ssuis*_ IgG antibodies are of interest for optimization of an rIde_*Ssuis*_-based vaccine. We speculate that important epitopes are in the conserved IgM protease domain. In the case of the mutant 10Δ*ide*_*Ssuis*__C-terminus, this domain is still expressed. Since this mutant lacks the C-terminal membrane anchor, the IgM protease domain is not linked to the streptococcal surface but secreted into the supernatant [9]. Accordingly, αIde_*Ssuis*_ IgG binding to the IgM protease domain are not expected to induce IgG-Fc_γ_ mediated-phagocytosis of 10Δ*ide*_*Ssuis*__C-terminus. This might explain why the mutant 10Δ*ide*_*Ssuis*__C-terminus shows significantly increased survival in blood of 4-week-old piglets in comparison with the wt, though both strains express functional IgM protease. Of note, this mutant is not attenuated in survival of blood of 8-week-old piglets carrying lower levels of αIde_*Ssuis*_ IgG [9].

It has been suggested that induction of “IgG1” [42] or a balanced ratio of “IgG1” to “IgG2” [43] is important for protection. However, the IgG subtype specificities of the respective reagents are unclear. Nevertheless, it is a limitation of this study that the ELISA used for determination of αIde_*Ssuis*_ IgG levels did not differentiate between different IgG subtypes. We envision that as soon as specific antibodies against each porcine IgG subtype are broadly available, it will be possible to read out levels of IgG subtypes that induce opsonophagoctosis via Fc_γ_ interaction on neutrophilic granulocytes. This might be crucial to define a specific level of αIde_*Ssuis*_ IgG as protective. As soon as an rIde_*Ssuis*_-based vaccine is available in the field, ELISA would be an important tool in porcine health management. Nevertheless, the LMM analysis already confirmed that αIde_*Ssuis*_ IgG levels as measured in this study have an effect on survival of *S. suis* *cps* 2 in porcine blood drawn from piglets in the field.

## Supplementary Information

**Additional file 1:** **Results of linear mixed-effects models for all investigated parameters on survival of** ***S. suis*** ***cps* 2 strain 10, ΔIde**_**Ssuis**_ **and ∇ide**_***Ssuis***_**_C195S in blood of piglets drawn at 2, 6 and 10 weeks of age**.

## Data Availability

Primary data of the longitudinal field study is available in the open research repository Zenodo [45]. All other primary data and materials will be made available on reasonable request.
