# Supplementary material for: Maternal antibodies against the immunoglobulin M-degrading enzyme of Streptococcus suis, IdeSsuis, play an important role in bactericidal immunity in young piglets in the field
Source: Vet Res. 2026 Mar 19;57:49. doi: 10.1186/s13567-026-01713-1 (PMC13064264; doi:10.1186/s13567-026-01713-1)
Supplement: Supplementary file 1 — Additional file 1: Results of linear mixed-effects models for all investigated parameters on survival of S. suis cps 2 strain 10, ΔIdeSsuis and ∇ideSsuis_C195S in blood of piglets drawn at 2, 6 and 10 weeks of age. [file 13567_2026_1713_MOESM1_ESM.pdf]

Supplementary material 1

Results of linear mixed-effects models for all investigated parameters on survival of *S. suis cps*2 strain 10, ΔIde$_{Ssuis}$ and ∇ide$_{Ssuis}$_C195S in blood of piglets drawn at 2, 6 and 10 weeks of age

Table of Contents

1. Strain S. suis 10 2
    - 1.1 Litter Order 2
    - 1.2 Sow Parity 3
    - 1.3 Piglet Birth Weight (Day0) [kg] 4
    - 1.4 Piglet Weight after 24 Hours (Day1) [kg] 4
    - 1.5 Piglet Weight in 2nd to 10th Postnatal Week (Weight) [kg] 5
    - 1.6 Colostrum Intake During the First 24 Hours (CI24) [kg] 6
    - 1.7 IgG Colostrum [log ELISA Units] 7
    - 1.8 IgG Serum [log ELISA Units] 8
    - 1.9 IgM Serum [log ELISA Units] 8
2. Strain S. suis ΔIdeSsuis 10
    - 2.1 Litter Order 10
    - 2.2 Sow Parity 11
    - 2.3 Piglet Birth Weight (Day0) [kg] 12
    - 2.4 Piglet Weight after 24 Hours (Day1) [kg] 12
    - 2.5 Piglet Weight in 2nd to 10th Postnatal Week (Weight) [kg] 13
    - 2.6 Colostrum Intake During the First 24 Hours (CI24) [kg] 14
    - 2.7 IgG Colostrum [log ELISA Units] 15
    - 2.8 IgG Serum [log ELISA Units] 16
    - 2.9 IgM Serum [log ELISA Units] 16
3. Strain S. suis ∇ide$_{Ssuis}$_C195S 18
    - 3.1 Litter Order 18
    - 3.2 Sow Parity 19
    - 3.3 Piglet Birth Weight (Day0) [kg] 20
    - 3.4 Piglet Weight after 24 Hours (Day1) [kg] 20
    - 3.5 Piglet Weight in 2nd to 10th Postnatal Week (Weight) [kg] 21
    - 3.6 Colostrum Intake During the First 24 Hours (CI24) [kg] 22
    - 3.7 IgG Colostrum [log ELISA Units] 23
    - 3.8 IgG Serum [log ELISA Units] 24
    - 3.9 IgM Serum [log ELISA Units] 24

1. Strain S. suis 10

M1 (Default Model):

Random effects:

Group	n	Parameter	Variance	Std. Dev.
Piglet:Sow	70	(Intercept)<br>Time	0.0080<br>0.0114	0.0892<br>0.1068
Sow	24	(Intercept)<br>Time	1.0543<br>0.0334	1.0268<br>0.1826
Residual			1.3668	1.1691

Fixed effects:

Parameter	Estimate β	95% CI	Std. Error	P value
(Intercept)	-1.0003	[-1.5290, -0.4739]	0.2696	**0.0012**
Time	-0.1189	[-0.2096, -0.0272]	0.0466	**0.0178**

1.1 Litter Order

M2 (Main Effect Model):

Random effects:

Group	n	Parameter	Variance	Std. Dev.
Piglet:Sow	70	(Intercept)<br>Time	0.0047<br>0.0108	0.0687<br>0.1037
Sow	24	(Intercept)<br>Time	1.0398<br>0.0334	1.0197<br>0.1828
Residual			1.3813	1.1753

Fixed effects:

Parameter	Estimate β	95% CI	Std. Error	P value
(Intercept)	-1.0050	[-1.5304, -0.4763]	0.2690	**0.0011**
Time	-0.1188	[-0.2101, -0.0278]	0.0466	**0.0179**
Litter Order (L)*	-0.1699	[-0.5160, 0.1790]	0.1758	0.3376
Litter Order (Q)*	-0.0419	[-0.3831, 0.2977]	0.1729	0.8096

M3 (Interaction Model):

Random effects:

Group	n	Parameter	Variance	Std. Dev.
Piglet:Sow	70	(Intercept)<br>Time	0.0000<br>0.0091	0.0000<br>0.0955
Sow	24	(Intercept)<br>Time	1.0495<br>0.0343	1.0245<br>0.1851
Residual			1.3905	1.1792

Fixed effects:

Parameter	Estimate β	95% CI	Std. Error	P value
(Intercept)	-0.9992	[-1.5237, -0.4695]	0.2701	**0.0012**
Time	-0.1201	[-0.2118, -0.0289]	0.0468	**0.0173**
Litter Order (L)*	-0.0461	[-0.6326, 0.5392]	0.2987	0.8776
Litter Order (Q)*	0.1384	[-0.4349, 0.7130]	0.2934	0.6381

Time:Litter Order (L)*	-0.0252	[-0.1197, 0.0692]	0.0482	0.6021
Time:Litter Order (Q)*	-0.0366	[-0.1288, 0.0559]	0.0473	0.4405

Model Comparison:

**Model**	**npar**	**Marginal R^2**	**Conditional R^2**	**Log-Likelihood**	**P value**
M1	9	0.0621	0.4398	-370.22	M2 vs M1: 0.6054
M2	11	0.0657	0.4375	-369.71	M3 vs M1: 0.7676
M3	13	0.0700	0.4396	-369.30	M3 vs M2: 0.6628

1.2 Sow Parity

M2 (Main Effect Model):

Random effects:

**Group**	**n**	**Parameter**	**Variance**	**Std. Dev.**
Piglet:Sow	70	(Intercept)<br>Time	0.0078<br>0.0114	0.0883<br>0.1068
Sow	24	(Intercept)<br>Time	1.0846<br>0.0333	1.0414<br>0.1826
Residual			1.3672	1.1693

Fixed effects:

**Parameter**	**Estimate β**	**95% CI**	**Std. Error**	**P value**
(Intercept)	-1.0002	[-1.5327, -0.4656]	0.2720	**0.0013**
Time	-0.1189	[-0.2102, -0.0277]	0.0466	**0.0178**
Sow Parity (L)*	-0.0852	[-0.5293, 0.3559]	0.2212	0.7043
Sow Parity (Q)*	-0.0478	[-0.4959, 0.4042]	0.2223	0.8318

M3 (Interaction Model):

Random effects:

**Group**	**n**	**Parameter**	**Variance**	**Std. Dev.**
Piglet:Sow	70	(Intercept)<br>Time	0.0076<br>0.0114	0.0872<br>0.1066
Sow	24	(Intercept)<br>Time	1.1925<br>0.0375	1.0920<br>0.1937
Residual			1.3670	1.1692

Fixed effects:

**Parameter**	**Estimate β**	**95% CI**	**Std. Error**	**P value**
(Intercept)	-0.9986	[-1.5511, -0.4506]	0.2801	**0.0018**
Time	-0.1192	[-0.2137, -0.0244]	0.0484	**0.0225**
Sow Parity (L)*	0.0467	[-0.9071, 0.9953]	0.4844	0.9241
Sow Parity (Q)*	-0.2726	[-1.2245, 0.6745]	0.4860	0.5807
Time:Sow Parity (L)*	-0.0256	[-0.1907, 0.1397]	0.0837	0.7630
Time:Sow Parity (Q)*	0.0436	[-0.1212, 0.2085]	0.0839	0.6085

Model Comparison:

**Model**	**npar**	**Marginal R^2**	**Conditional R^2**	**Log-Likelihood**	**P value**
M1	9	0.0621	0.4398	-370.22	M2 vs M1: 0.8870
M2	11	0.0625	0.4472	-370.10	M3 vs M1: 0.9578
M3	13	0.0649	0.4595	-369.89	M3 vs M2: 0.8160

*Since Litter Order and Sow Parity are ordinal variables with three levels, two orthogonal polynomial contrasts (one fewer than the number of levels) are fitted to the levels. While the first contrast (L) is linear, the second contrast (Q) is quadratic.

1.3 Piglet Birth Weight (Day0) [kg]

M2 (Main Effect Model):

Random effects:

Group	n	Parameter	Variance	Std. Dev.
Piglet:Sow	69	(Intercept)<br>Time	0.0336<br>0.0126	0.1833<br>0.1123
Sow	24	(Intercept)<br>Time	1.1658<br>0.0341	1.0797<br>0.1847
Residual			1.2324	1.1101

Fixed effects:

Parameter	Estimate β	95% CI	Std. Error	P value
(Intercept)	-0.6132	[-1.9258, 0.6942]	0.6553	0.3523
Time	-0.1161	[-0.2077, -0.0242]	0.0466	**0.0204**
Day0	-0.2602	[-1.1196, 0.6017]	0.4281	0.5454

M3 (Interaction Model):

Random effects:

Group	n	Parameter	Variance	Std. Dev.
Piglet:Sow	69	(Intercept)<br>Time	0.0395<br>0.0132	0.1986<br>0.1150
Sow	24	(Intercept)<br>Time	1.1508<br>0.0335	1.0728<br>0.1830
Residual			1.2378	1.1126

Fixed effects:

Parameter	Estimate β	95% CI	Std. Error	P value
(Intercept)	-1.0563	[-3.4552, 1.3254]	1.2025	0.382
Time	-0.0292	[-0.4338, 0.3769]	0.2029	0.886
Day0	0.0587	[-1.6158, 1.7380]	0.8430	0.945
Time:Day0	-0.0626	[-0.3479, 0.2206]	0.1422	0.661

Model Comparison:

Model	npar	Marginal R^2	Conditional R^2	Log-Likelihood	P value
M1	9	0.0638	0.4556	-355.60	M2 vs M1: 0.5417
M2	10	0.0655	0.4575	-355.41	M3 vs M1: 0.7446
M3	11	0.0676	0.4574	-355.31	M3 vs M2: 0.6410

1.4 Piglet Weight after 24 Hours (Day1) [kg]

M2 (Main Effect Model):

Random effects:

Group	n	Parameter	Variance	Std. Dev.
Piglet:Sow	69	(Intercept)<br>Time	0.0351<br>0.0127	0.1875<br>0.1128
Sow	24	(Intercept)<br>Time	1.1675<br>0.0341	1.0805<br>0.1847

Residual			1.2299	1.1090

Fixed effects:

Parameter	Estimate β	95% CI	Std. Error	P value
(Intercept)	-0.4362	[-1.7113, 0.8327]	0.6353	0.4942
Time	-0.1160	[-0.2074, -0.0248]	0.0466	**0.0204**
Day1	-0.3636	[-1.1403, 0.4138]	0.3865	0.3501

M3 (Interaction Model):

Random effects:

Group	n	Parameter	Variance	Std. Dev.
Piglet:Sow	69	(Intercept)<br>Time	0.0402<br>0.0132	0.2005<br>0.1149
Sow	24	(Intercept)<br>Time	1.1681<br>0.0341	1.0808<br>0.1847
Residual			1.2347	1.1112

Fixed effects:

Parameter	Estimate β	95% CI	Std. Error	P value
(Intercept)	-0.6767	[-2.9709, 1.6154]	1.1525	0.558
Time	-0.0688	[-0.4580, 0.3179]	0.1945	0.724
Day1	-0.2011	[-1.7027, 1.3087]	0.7558	0.791
Time:Day1	-0.0319	[-0.2867, 0.2215]	0.1275	0.803

Model Comparison:

Model	npar	Marginal R^2	Conditional R^2	Log-Likelihood	P value
M1	9	0.0638	0.4556	-355.60	M2 vs M1: 0.6475
M2	10	0.0680	0.4585	-355.50	M3 vs M1: 0.6168
M3	11	0.0689	0.4594	-355.12	M3 vs M2: 0.3842

1.5 Piglet Weight in 2nd to 10th Postnatal Week (Weight) [kg]

M2 (Main Effect Model):

Random effects:

Group	n	Parameter	Variance	Std. Dev.
Piglet:Sow	69	(Intercept)<br>Time	0.0436<br>0.0135	0.2087<br>0.1163
Sow	24	(Intercept)<br>Time	1.1553<br>0.0353	1.0748<br>0.1878
Residual			1.2168	1.1031

Fixed effects:

Parameter	Estimate β	95% CI	Std. Error	P value
(Intercept)	-1.0367	[-1.5856, -0.4861]	0.2799	**0.0010**
Time	-0.0494	[-0.2074, 0.1097]	0.0803	0.5391
Weight	-0.0266	[-0.0777, 0.0249]	0.0259	0.3061

M3 (Interaction Model):

Random effects:

Group	n	Parameter	Variance	Std. Dev.
Piglet:Sow	69	(Intercept)<br>Time	0.0334<br>0.0128	0.1826<br>0.1130
Sow	24	(Intercept)<br>Time	1.1384<br>0.0345	1.0669<br>0.1858
Residual			1.2256	1.1070

Fixed effects:

Parameter	Estimate β	95% CI	Std. Error	P value
(Intercept)	-1.4768	[-2.4965, -0.4622]	0.5104	**0.0044**
Time	-0.0637	[-0.2236, 0.0963]	0.0810	0.4335
Weight	0.0939	[-0.1456, 0.3341]	0.1189	0.4311
Time:Weight	-0.0098	[-0.0288, 0.0091]	0.0095	0.3035

Model Comparison:

Model	npar	Marginal R^2	Conditional R^2	Log-Likelihood	P value
M1	9	0.0638	0.4556	-355.60	M2 vs M1: 0.3202
M2	10	0.0668	0.4691	-355.11	M3 vs M1: 0.3533
M3	11	0.0690	0.4628	-354.56	M3 vs M2: 0.2959

1.6 Colostrum Intake During the First 24 Hours (CI24) [kg]

M2 (Main Effect Model):

Random effects:

Group	n	Parameter	Variance	Std. Dev.
Piglet:Sow	69	(Intercept)<br>Time	0.0359<br>0.0126	0.1896<br>0.1124
Sow	24	(Intercept)<br>Time	1.1188<br>0.0342	1.0577<br>0.1848
Residual			1.2302	1.1092

Fixed effects:

Parameter	Estimate β	95% CI	Std. Error	P value
(Intercept)	-0.4384	[-1.4532, 0.5810]	0.5099	0.3921
Time	-0.1160	[-0.2071, -0.0242]	0.0466	**0.0204**
CI24	-1.1969	[-3.1512, 0.7257]	0.9625	0.2171

M3 (Interaction Model):

Random effects:

Group	n	Parameter	Variance	Std. Dev.
Piglet:Sow	69	(Intercept)<br>Time	0.0406<br>0.0131	0.2015<br>0.1144
Sow	24	(Intercept)<br>Time	1.1273<br>0.0345	1.0617<br>0.1856
Residual			1.2344	1.1110

Fixed effects:

Parameter	Estimate β	95% CI	Std. Error	P value
(Intercept)	-0.3472	[-2.0767, 1.3691]	0.8650	0.689
Time	-0.1341	[-0.4255, 0.1597]	0.1466	0.363
CI24	-1.4002	[-5.0592, 2.2699]	1.8310	0.446
Time:CI24	0.0404	[-0.5815, 0.6575]	0.3097	0.897

Model Comparison:

Model	npar	Marginal R^2	Conditional R^2	Log-Likelihood	P value
M1	9	0.0638	0.4556	-355.60	M2 vs M1: 0.2090
M2	10	0.0707	0.4591	-354.81	M3 vs M1: 0.4501
M3	11	0.0699	0.4600	-354.80	M3 vs M2: 0.8934

1.7 IgG Colostrum [log ELISA Units]

M2 (Main Effect Model):

Random effects:

Group	n	Parameter	Variance	Std. Dev.
Piglet:Sow	69	(Intercept)<br>Time	0.0350<br>0.0125	0.1871<br>0.1120
Sow	24	(Intercept)<br>Time	1.1430<br>0.0342	1.0691<br>0.1849
Residual			1.2322	1.1100

Fixed effects:

Parameter	Estimate β	95% CI	Std. Error	P value
(Intercept)	-1.1731	[-2.7036, 0.3627]	0.7670	0.1386
Time	-0.1161	[-0.2075, -0.0244]	0.0466	**0.0204**
IgG Colostrum	0.0687	[-0.4328, 0.5673]	0.2489	0.7852

M3 (Interaction Model):

Random effects:

Group	n	Parameter	Variance	Std. Dev.
Piglet:Sow	69	(Intercept)<br>Time	0.0348<br>0.0125	0.1866<br>0.1119
Sow	24	(Intercept)<br>Time	1.2082<br>0.0366	1.0992<br>0.1913
Residual			1.2322	1.1101

Fixed effects:

Parameter	Estimate β	95% CI	Std. Error	P value
(Intercept)	-1.2344	[-4.4298, 2.0332]	1.6479	0.462
Time	-0.1043	[-0.6608, 0.4500]	0.2829	0.716
IgG Colostrum	0.0901	[-1.0277, 1.1854]	0.5640	0.874
Time:IgG Colostrum	-0.0041	[-0.1932, 0.1867]	0.0968	0.967

Model Comparison:

Model	npar	Marginal R^2	Conditional R^2	Log-Likelihood	P value
M1	9	0.0638	0.4556	-355.60	M2 vs M1: 0.7765
M2	10	0.0638	0.4596	-355.56	M3 vs M1: 0.9594

M3	11	0.0631	0.4659	-355.56	M3 vs M2: 0.9625

1.8 IgG Serum [log ELISA Units]

M2 (Main Effect Model):

Random effects:

Group	n	Parameter	Variance	Std. Dev.
Piglet:Sow	69	(Intercept)<br>Time	0.0184<br>0.0113	0.1357<br>0.1063
Sow	24	(Intercept)<br>Time	1.4399<br>0.0385	1.2000<br>0.1963
Residual			1.1426	1.0689

Fixed effects:

Parameter	Estimate β	95% CI	Std. Error	P value
(Intercept)	0.3417	[-0.6108, 1.3020]	0.4796	0.4782
Time	-0.1860	[-0.2885, -0.0833]	0.0520	**0.0012**
IgG Serum	-0.9723	[-1.5410, -0.4060]	0.2821	**<0.001**

M3 (Interaction Model):

Random effects:

Group	n	Parameter	Variance	Std. Dev.
Piglet:Sow	69	(Intercept)<br>Time	0.0093<br>0.0104	0.0966<br>0.1021
Sow	24	(Intercept)<br>Time	1.3352<br>0.0361	1.1555<br>0.1899
Residual			1.1429	1.0691

Fixed effects:

Parameter	Estimate β	95% CI	Std. Error	P value
(Intercept)	-0.1320	[-1.3079, 1.0554]	0.5910	0.824
Time	-0.0923	[-0.2659, 0.0811]	0.0870	0.291
IgG Serum	-0.5186	[-1.4052, 0.3660]	0.4411	0.242
Time:IgG Serum	-0.1063	[-0.2677, 0.0554]	0.0806	0.189

Model Comparison:

Model	npar	Marginal R^2	Conditional R^2	Log-Likelihood	P value
M1	9	0.0638	0.4556	-355.60	M2 vs M1: **<0.001**
M2	10	0.1124	0.5047	-350.09	M3 vs M1: **0.0017**
M3	11	0.1253	**0.5065**	-349.22	M3 vs M2: 0.1883

1.9 IgM Serum [log ELISA Units]

M2 (Main Effect Model):

Random effects:

Group	n	Parameter	Variance	Std. Dev.
Piglet:Sow	69	(Intercept)<br>Time	0.0335<br>0.0123	0.1830<br>0.1107
Sow	24	(Intercept)<br>Time	1.1443<br>0.0342	1.0697<br>0.1848
Residual			1.2416	1.1143

Fixed effects:

Parameter	Estimate β	95% CI	Std. Error	P value
(Intercept)	-0.9635	[-1.5017, -0.4237]	0.2747	**0.0018**
Time	-0.0983	[-0.2232, 0.0270]	0.0633	0.1253
IgM Serum	-0.0372	[-0.2146, 0.1407]	0.0896	0.6785

M3 (Interaction Model):

Random effects:

Group	n	Parameter	Variance	Std. Dev.
Piglet:Sow	69	(Intercept)<br>Time	0.0191<br>0.0104	0.1383<br>0.1020
Sow	24	(Intercept)<br>Time	1.0872<br>0.0308	1.0427<br>0.1753
Residual			1.2168	1.1031

Fixed effects:

Parameter	Estimate β	95% CI	Std. Error	P value
(Intercept)	-1.7335	[-2.5293, -0.9378]	0.4012	**<0.001**
Time	0.2499	[-0.0455, 0.5476]	0.1473	0.0923
IgM Serum	0.1278	[-0.0889, 0.3442]	0.1101	0.2475
Time:IgM Serum	-0.0762	[-0.1352, -0.0174]	0.0294	**0.0106**

Model Comparison:

Model	npar	Marginal R^2	Conditional R^2	Log-Likelihood	P value
M1	9	0.0638	0.4556	-355.60	M2 vs M1: 0.6843
M2	10	0.0642	0.4525	-355.52	M3 vs M1: **0.0343**
M3	11	0.1011	0.4656	-352.23	M3 vs M2: **0.0103**

2. Strain S. suis ΔIdeSsuis

M1 (Default Model):

Random effects:

Group	n	Parameter	Variance	Std. Dev.
Piglet:Sow	70	(Intercept)<br>Time	0.0000<br>0.0061	0.0002<br>0.0780
Sow	24	(Intercept)<br>Time	1.4490<br>0.0343	1.2038<br>0.1851
Residual			1.6920	1.3007

Fixed effects:

Parameter	Estimate β	95% CI	Std. Error	P value
(Intercept)	0.1937	[-0.4137, 0.7980]	0.3096	0.5377
Time	-0.2052	[-0.2982, -0.1117]	0.0477	**<0.001**

2.1 Litter Order

M2 (Main Effect Model):

Random effects:

Group	n	Parameter	Variance	Std. Dev.
Piglet:Sow	70	(Intercept)<br>Time	0.0000<br>0.0062	0.0000<br>0.0786
Sow	24	(Intercept)<br>Time	1.4381<br>0.0341	1.1992<br>0.1847
Residual			1.7034	1.3051

Fixed effects:

Parameter	Estimate β	95% CI	Std. Error	P value
(Intercept)	0.1898	[-0.4161, 0.7985]	0.3093	0.5454
Time	-0.2051	[-0.2984, -0.1115]	0.0477	**<0.001**
Litter Order (L)*	-0.1699	[-0.5329, 0.1938]	0.1835	0.3580
Litter Order (Q)*	0.0283	[-0.3263, 0.3843]	0.1803	0.8758

M3 (Interaction Model):

Random effects:

Group	n	Parameter	Variance	Std. Dev.
Piglet:Sow	70	(Intercept)<br>Time	0.0000<br>0.0063	0.0000<br>0.0792
Sow	24	(Intercept)<br>Time	1.4482<br>0.0343	1.2034<br>0.1852
Residual			1.7124	1.3086

Fixed effects:

Parameter	Estimate β	95% CI	Std. Error	P value
(Intercept)	0.1971	[-0.4066, 0.8060]	0.3104	0.5317
Time	-0.2065	[-0.3004, -0.1132]	0.0479	**<0.001**
Litter Order (L)*	-0.0041	[-0.6546, 0.6469]	0.3317	0.9903
Litter Order (Q)*	0.2328	[-0.4032, 0.8704]	0.3256	0.4762
Time:Litter Order (L)*	-0.0308	[-0.1314, 0.0694]	0.0513	0.5499
Time:Litter Order (Q)*	-0.0380	[-0.1365, 0.0604]	0.0504	0.4526

Model Comparison:

Model	npar	Marginal R^2	Conditional R^2	Log-Likelihood	P value
M1	9	0.1505	0.4356	-385.39	M2 vs M1: 0.6335
M2	11	0.1528	0.4353	-384.93	M3 vs M1: 0.7671
M3	13	0.1553	0.4368	-384.47	M3 vs M2: 0.6323

2.2 Sow Parity

M2 (Main Effect Model):

Random effects:

Group	N	Parameter	Variance	Std. Dev.
Piglet:Sow	70	(Intercept)<br>Time	0.0000<br>0.0061	0.0000<br>0.0779
Sow	24	(Intercept)<br>Time	1.5025<br>0.0343	1.2258<br>0.1851
Residual			1.6920	1.3008

Fixed effects:

Parameter	Estimate β	95% CI	Std. Error	P value
(Intercept)	0.1933	[-0.4195, 0.8085]	0.3132	0.5431
Time	-0.2052	[-0.2988, -0.1116]	0.0477	**<0.001**
Sow Parity (L)*	-0.1476	[-0.6522, 0.3569]	0.2516	0.5643
Sow Parity (Q)*	0.0255	[-0.4838, 0.5308]	0.2527	0.9208

M3 (Interaction Model):

Random effects:

Group	n	Parameter	Variance	Std. Dev.
Piglet:Sow	70	(Intercept)<br>Time	0.0000<br>0.0060	0.0000<br>0.0776
Sow	24	(Intercept)<br>Time	1.6486<br>0.0388	1.2840<br>0.1970
Residual			1.6917	1.3007

Fixed effects:

Parameter	Estimate β	95% CI	Std. Error	P value
(Intercept)	0.1950	[-0.4414, 0.8262]	0.3228	0.5522
Time	-0.2055	[-0.3024, -0.1080]	0.0496	**<0.001**
Sow Parity (L)*	-0.0109	[-1.1080, 1.0828]	0.5583	0.9846
Sow Parity (Q)*	-0.2385	[-1.3359, 0.8535]	0.5599	0.6744
Time:Sow Parity (L)*	-0.0235	[-0.1932, 0.1462]	0.0859	0.7874
Time:Sow Parity (Q)*	0.0455	[-0.1235, 0.2151]	0.0861	0.6030

Model Comparison:

Model	npar	Marginal R^2	Conditional R^2	Log-Likelihood	P value
M1	9	0.1505	0.4356	-385.39	M2 vs M1: 0.8089
M2	11	0.1509	0.4435	-385.18	M3 vs M1: 0.9362
M3	13	0.1511	0.4538	-384.98	M3 vs M2: 0.8218

*Since Litter Order and Sow Parity are ordinal variables with three levels, two orthogonal polynomial contrasts (one fewer than the number of levels) are fitted to the levels. While the first contrast (L) is linear, the second contrast (Q) is quadratic.

2.3 Piglet Birth Weight (Day0) [kg]

M2 (Main Effect Model):

Random effects:

Group	n	Parameter	Variance	Std. Dev.
Piglet:Sow	69	(Intercept)<br>Time	0.0002<br>0.0051	0.0143<br>0.0717
Sow	24	(Intercept)<br>Time	1.5068<br>0.0345	1.2275<br>0.1857
Residual			1.6753	1.2943

Fixed effects:

Parameter	Estimate β	95% CI	Std. Error	P value
(Intercept)	0.5024	[-1.0086, 2.0024]	0.7498	0.5048
Time	-0.2010	[-0.2942, -0.1072]	0.0477	**<0.001**
Day0	-0.2205	[-1.2099, 0.7619]	0.4901	0.6543

M3 (Interaction Model):

Random effects:

Group	n	Parameter	Variance	Std. Dev.
Piglet:Sow	69	(Intercept)<br>Time	0.0004<br>0.0053	0.0205<br>0.0728
Sow	24	(Intercept)<br>Time	1.5540<br>0.0358	1.2466<br>0.1893
Residual			1.6788	1.2957

Fixed effects:

Parameter	Estimate β	95% CI	Std. Error	P value
(Intercept)	0.7181	[-2.0868, 3.4925]	1.3918	0.607
Time	-0.2391	[-0.6656, 0.1904]	0.2130	0.265
Day0	-0.3757	[-2.3358, 1.5868]	0.9758	0.701
Time:Day0	0.0274	[-0.2750, 0.3269]	0.1493	0.855

Model Comparison:

Model	npar	Marginal R^2	Conditional R^2	Log-Likelihood	P value
M1	9	0.1475	0.4281	-377.81	M2 vs M1: 0.6642
M2	10	0.1476	0.4328	-377.72	M3 vs M1: 0.8992
M3	11	0.1467	0.4351	-377.71	M3 vs M2: 0.8771

2.4 Piglet Weight after 24 Hours (Day1) [kg]

M2 (Main Effect Model):

Random effects:

Group	n	Parameter	Variance	Std. Dev.
Piglet:Sow	69	(Intercept)<br>Time	0.0005<br>0.0052	0.0217<br>0.0721
Sow	24	(Intercept)<br>Time	1.5081<br>0.0345	1.2280<br>0.1858
Residual			1.6720	1.2931

Fixed effects:

Parameter	Estimate β	95% CI	Std. Error	P value
(Intercept)	0.6489	[-0.8095, 2.0963]	0.7266	0.3743
Time	-0.2010	[-0.2948, -0.1070]	0.0477	**<0.001**
Day1	-0.3057	[-1.1907, 0.5858]	0.4424	0.4918

M3 (Interaction Model):

Random effects:

Group	n	Parameter	Variance	Std. Dev.
Piglet:Sow	69	(Intercept)<br>Time	0.0008<br>0.0054	0.0285<br>0.0736
Sow	24	(Intercept)<br>Time	1.5617<br>0.0358	1.2497<br>0.1891
Residual			1.6737	1.2937

Fixed effects:

Parameter	Estimate β	95% CI	Std. Error	P value
(Intercept)	1.0274	[-1.6178, 3.6822]	1.3320	0.442
Time	-0.2680	[-0.6747, 0.1404]	0.2038	0.192
Day1	-0.5611	[-2.3011, 1.1837]	0.8736	0.522
Time:Day1	0.0453	[-0.2231, 0.3126]	0.1337	0.736

Model Comparison:

Model	npar	Marginal R^2	Conditional R^2	Log-Likelihood	P value
M1	9	0.1475	0.4281	-377.81	M2 vs M1: 0.5053
M2	10	0.1488	0.4347	-377.59	M3 vs M1: 0.7637
M3	11	0.1480	0.4374	-377.54	M3 vs M2: 0.7577

2.5 Piglet Weight in 2nd to 10th Postnatal Week (Weight) [kg]

M2 (Main Effect Model):

Random effects:

Group	n	Parameter	Variance	Std. Dev.
Piglet:Sow	69	(Intercept)<br>Time	0.0011<br>0.0054	0.0333<br>0.0736
Sow	24	(Intercept)<br>Time	1.4823<br>0.0348	1.2175<br>0.1866
Residual			1.6833	1.2974

Fixed effects:

Parameter	Estimate β	95% CI	Std. Error	P value
(Intercept)	0.1623	[-0.4675, 0.7901]	0.3196	0.6159
Time	-0.1650	[-0.3355, 0.0070]	0.0866	0.0591
Weight	-0.0144	[-0.0713, 0.0426]	0.0287	0.6181

M3 (Interaction Model):

Random effects:

Group	n	Parameter	Variance	Std. Dev.
Piglet:Sow	69	(Intercept)<br>Time	0.0002<br>0.0052	0.0140<br>0.0719
Sow	24	(Intercept)<br>Time	1.4825<br>0.0348	1.2176<br>0.1866
Residual			1.6890	1.2996

Fixed effects:

Parameter	Estimate β	95% CI	Std. Error	P value
(Intercept)	-0.0231	[-1.1883, 1.1315]	0.5852	0.9685
Time	-0.1683	[-0.3399, 0.0030]	0.0870	0.0553
Weight	0.0347	[-0.2270, 0.2997]	0.1318	0.7925
Time:Weight	-0.0041	[-0.0256, 0.0173]	0.0107	0.7047

Model Comparison:

Model	npar	Marginal R^2	Conditional R^2	Log-Likelihood	P value
M1	9	0.1475	0.4281	-377.81	M2 vs M1: 0.6221
M2	10	0.1478	0.4287	-377.69	M3 vs M1: 0.8241
M3	11	0.1476	0.4288	-377.62	M3 vs M2: 0.7043

2.6 Colostrum Intake During the First 24 Hours (CI24) [kg]

M2 (Main Effect Model):

Random effects:

Group	n	Parameter	Variance	Std. Dev.
Piglet:Sow	69	(Intercept)<br>Time	0.0000<br>0.0046	0.0045<br>0.0681
Sow	24	(Intercept)<br>Time	1.4720<br>0.0348	1.2131<br>0.1865
Residual			1.6730	1.2936

Fixed effects:

Parameter	Estimate β	95% CI	Std. Error	P value
(Intercept)	0.5802	[-0.5857, 1.7386]	0.5821	0.3212
Time	-0.2009	[-0.2951, -0.1077]	0.0478	**<0.001**
CI24	-0.8571	[-3.0636, 1.3407]	1.0962	0.4361

M3 (Interaction Model):

Random effects:

Group	n	Parameter	Variance	Std. Dev.
Piglet:Sow	69	(Intercept)<br>Time	0.0000<br>0.0048	0.0000<br>0.0694
Sow	24	(Intercept)<br>Time	1.4871<br>0.0344	1.2195<br>0.1856
Residual			1.6773	1.2951

Fixed effects:

Parameter	Estimate β	95% CI	Std. Error	P value
(Intercept)	1.0604	[-0.9400, 3.0665]	0.9992	0.2908
Time	-0.2880	[-0.5915, 0.0157]	0.1527	0.0619
CI24	-1.9278	[-6.2048, 2.3296]	2.1162	0.3640
Time:CI24	0.1941	[-0.4522, 0.8373]	0.3234	0.5495

Model Comparison:

Model	npar	Marginal R^2	Conditional R^2	Log-Likelihood	P value
M1	9	0.1475	0.4281	-377.81	M2 vs M1: 0.4559
M2	10	0.1489	0.4350	-377.53	M3 vs M1: 0.6271
M3	11	0.1494	0.4346	-377.35	M3 vs M2: 0.5390

2.7 IgG Colostrum [log ELISA Units]

M2 (Main Effect Model):

Random effects:

Group	n	Parameter	Variance	Std. Dev.
Piglet:Sow	69	(Intercept)<br>Time	0.0000<br>0.0047	0.0000<br>0.0684
Sow	24	(Intercept)<br>Time	1.4547<br>0.0347	1.2061<br>0.1863
Residual			1.6785	1.2956

Fixed effects:

Parameter	Estimate β	95% CI	Std. Error	P value
(Intercept)	-0.4633	[-2.2672, 1.3193]	0.8952	0.6095
Time	-0.2009	[-0.2943, -0.1065]	0.0478	**<0.001**
IgG Colostrum	0.2287	[-0.3548, 0.8156]	0.2915	0.4418

M3 (Interaction Model):

Random effects:

Group	n	Parameter	Variance	Std. Dev.
Piglet:Sow	69	(Intercept)<br>Time	0.0002<br>0.0049	0.0134<br>0.0701
Sow	24	(Intercept)<br>Time	1.5266<br>0.0368	1.2356<br>0.1918
Residual			1.6782	1.2955

Fixed effects:

Parameter	Estimate β	95% CI	Std. Error	P value
(Intercept)	-1.0515	[-4.6724, 2.6591]	1.8703	0.580
Time	-0.0989	[-0.6682, 0.4679]	0.2889	0.736
IgG Colostrum	0.4331	[-0.8378, 1.6738]	0.6401	0.506
Time:IgG Colostrum	-0.0355	[-0.2291, 0.1604]	0.0989	0.724

Model Comparison:

Model	npar	Marginal R^2	Conditional R^2	Log-Likelihood	P value
M1	9	0.1475	0.4281	-377.81	M2 vs M1: 0.4196
M2	10	0.1507	0.4327	-377.49	M3 vs M1: 0.6720
M3	11	0.1503	0.4377	-377.41	M3 vs M2: 0.7047

2.8 IgG Serum [log ELISA Units]

M2 (Main Effect Model):

Random effects:

Group	n	Parameter	Variance	Std. Dev.
Piglet:Sow	69	(Intercept)<br>Time	0.0000<br>0.0049	0.0018<br>0.0700
Sow	24	(Intercept)<br>Time	1.6250<br>0.0369	1.2746<br>0.1921
Residual			1.6510	1.2850

Fixed effects:

Parameter	Estimate β	95% CI	Std. Error	P value
(Intercept)	0.7093	[-0.3853, 1.8101]	0.5499	0.2005
Time	-0.2282	[-0.3350, -0.1213]	0.0541	**<0.001**
IgG Serum	-0.3792	[-1.0464, 0.2899]	0.3305	0.2528

M3 (Interaction Model):

Random effects:

Group	n	Parameter	Variance	Std. Dev.
Piglet:Sow	69	(Intercept)<br>Time	0.0000<br>0.0049	0.0000<br>0.0702
Sow	24	(Intercept)<br>Time	1.6461<br>0.0375	1.2830<br>0.1935
Residual			1.6577	1.2875

Fixed effects:

Parameter	Estimate β	95% CI	Std. Error	P value
(Intercept)	0.7599	[-0.6408, 2.1663]	0.7046	0.2836
Time	-0.2375	[-0.4321, -0.0424]	0.0979	**0.0168**
IgG Serum	-0.4266	[-1.4806, 0.6311]	0.5290	0.4213
Time:IgG Serum	0.0102	[-0.1700, 0.1900]	0.0903	0.9102

Model Comparison:

Model	npar	Marginal R^2	Conditional R^2	Log-Likelihood	P value
M1	9	0.1475	0.4281	-377.81	M2 vs M1: 0.2752
M2	10	0.1515	0.4452	-377.22	M3 vs M1: 0.5482
M3	11	0.1508	0.4456	-377.21	M3 vs M2: 0.9143

2.9 IgM Serum [log ELISA Units]

M2 (Main Effect Model):

Random effects:

Group	n	Parameter	Variance	Std. Dev.
Piglet:Sow	69	(Intercept)<br>Time	0.0001<br>0.0047	0.0106<br>0.0687
Sow	24	(Intercept)<br>Time	1.5075<br>0.0349	1.2278<br>0.1867
Residual			1.6855	1.2983

Fixed effects:

Parameter	Estimate β	95% CI	Std. Error	P value
(Intercept)	0.2137	[-0.4062, 0.8335]	0.3160	0.5054
Time	-0.1740	[-0.3097, -0.0384]	0.0688	**0.0135**
IgM Serum	-0.0565	[-0.2600, 0.1485]	0.1033	0.5853

M3 (Interaction Model):

Random effects:

Group	n	Parameter	Variance	Std. Dev.
Piglet:Sow	69	(Intercept)<br>Time	0.0000<br>0.0041	0.0000<br>0.0640
Sow	24	(Intercept)<br>Time	1.4464<br>0.0332	1.2027<br>0.1822
Residual			1.6870	1.2988

Fixed effects:

Parameter	Estimate β	95% CI	Std. Error	P value
(Intercept)	-0.1977	[-1.0873, 0.6950]	0.4505	0.662
Time	0.0068	[-0.3079, 0.3231]	0.1573	0.966
IgM Serum	0.0396	[-0.2152, 0.2961]	0.1292	0.760
Time:IgM Serum	-0.0404	[-0.1047, 0.0231]	0.0319	0.207

Model Comparison:

Model	npar	Marginal R^2	Conditional R^2	Log-Likelihood	P value
M1	9	0.1475	0.4281	-377.81	M2 vs M1: 0.6015
M2	10	0.1477	0.4285	-377.68	M3 vs M1: 0.4075
M3	11	0.1548	0.4327	-376.91	M3 vs M2: 0.2172

3. Strain S. suis ∇ide_{Ssuis}_C195S

M1 (Default Model):

Random effects:

Group	n	Parameter	Variance	Std. Dev.
Piglet:Sow	70	(Intercept)<br>Time	0.0000<br>0.0010	0.0035<br>0.0324
Sow	24	(Intercept)<br>Time	1.2100<br>0.0222	1.1000<br>0.1490
Residual			1.7535	1.3242

Fixed effects:

Parameter	Estimate β	95% CI	Std. Error	P value
(Intercept)	-0.1802	[-0.7582, 0.4001]	0.2953	0.5475
Time	-0.1737	[-0.2554, -0.0916]	0.0416	**<0.001**

3.1 Litter Order

M2 (Main Effect Model):

Random effects:

Group	n	Parameter	Variance	Std. Dev.
Piglet:Sow	70	(Intercept)<br>Time	0.0001<br>0.0006	0.0104<br>0.0252
Sow	24	(Intercept)<br>Time	1.1934<br>0.0222	1.0924<br>0.1491
Residual			1.7655	1.3287

Fixed effects:

Parameter	Estimate β	95% CI	Std. Error	P value
(Intercept)	-0.1873	[-0.7656, 0.3877]	0.2946	0.5310
Time	-0.1736	[-0.2554, -0.0922]	0.0416	**<0.001**
Litter Order (L)*	-0.2408	[-0.5642, 0.0838]	0.1643	0.1473
Litter Order (Q)*	-0.0654	[-0.3835, 0.2525]	0.1614	0.6868

M3 (Interaction Model):

Random effects:

Group	n	Parameter	Variance	Std. Dev.
Piglet:Sow	70	(Intercept)<br>Time	0.0000<br>0.0007	0.0033<br>0.0260
Sow	24	(Intercept)<br>Time	1.1960<br>0.0223	1.0936<br>0.1492
Residual			1.7800	1.3341

Fixed effects:

Parameter	Estimate β	95% CI	Std. Error	P value
(Intercept)	-0.1813	[-0.7608, 0.3994]	0.2954	0.5453
Time	-0.1746	[-0.2564, -0.0928]	0.0417	**<0.001**
Litter Order (L)*	-0.1128	[-0.7727, 0.5489]	0.3380	0.7391
Litter Order (Q)*	0.1401	[-0.5102, 0.7915]	0.3319	0.6738
Time:Litter Order (L)*	-0.0216	[-0.1190, 0.0756]	0.0498	0.6653
Time:Litter Order (Q)*	-0.0347	[-0.1301, 0.0609]	0.0489	0.4796

Model Comparison:

Model	npar	Marginal R^2	Conditional R^2	Log-Likelihood	P value
M1	9	0.1263	0.3148	-375.63	M2 vs M1: 0.3168
M2	11	0.1334	0.3143	-374.48	M3 vs M1: 0.5608
M3	13	0.1350	0.3139	-374.14	M3 vs M2: 0.7107

3.2 Sow Parity

M2 (Main Effect Model):

Random effects:

Group	N	Parameter	Variance	Std. Dev.
Piglet:Sow	70	(Intercept)<br>Time	0.0000<br>0.0011	0.0019<br>0.0324
Sow	24	(Intercept)<br>Time	1.2610<br>0.0222	1.1231<br>0.1489
Residual			1.7540	1.3245

Fixed effects:

Parameter	Estimate β	95% CI	Std. Error	P value
(Intercept)	-0.1810	[-0.7682, 0.4109]	0.2989	0.5507
Time	-0.1738	[-0.2558, -0.0917]	0.0416	**<0.001**
Sow Parity (L)*	-0.1805	[-0.6409, 0.2791]	0.2298	0.4415
Sow Parity (Q)*	0.1176	[-0.3513, 0.5804]	0.2308	0.6159

M3 (Interaction Model):

Random effects:

Group	n	Parameter	Variance	Std. Dev.
Piglet:Sow	70	(Intercept)<br>Time	0.0000<br>0.0010	0.0051<br>0.0319
Sow	24	(Intercept)<br>Time	1.4070<br>0.0257	1.1862<br>0.1604
Residual			1.7540	1.3243

Fixed effects:

Parameter	Estimate β	95% CI	Std. Error	P value
(Intercept)	-0.1791	[-0.7840, 0.4288]	0.3089	0.5682
Time	-0.1741	[-0.2586, -0.0895]	0.0433	**<0.001**
Sow Parity (L)*	0.0031	[-1.0416, 1.0474]	0.5341	0.9955
Sow Parity (Q)*	-0.0420	[-1.0892, 1.0049]	0.5359	0.9383
Time:Sow Parity (L)*	-0.0284	[-0.1752, 0.1182]	0.0749	0.7078
Time:Sow Parity (Q)*	0.0248	[-0.1211, 0.1723]	0.0751	0.7446

Model Comparison:

Model	npar	Marginal R^2	Conditional R^2	Log-Likelihood	P value
M1	9	0.1263	0.3148	-375.63	M2 vs M1: 0.6095
M2	11	0.1306	0.3245	-375.13	M3 vs M1: 0.8653
M3	13	0.1296	0.3350	-374.99	M3 vs M2: 0.8666

*Since Litter Order and Sow Parity are ordinal variables with three levels, two orthogonal polynomial contrasts (one fewer than the number of levels) are fitted to the levels. While the first contrast (L) is linear, the second contrast (Q) is quadratic.

3.3 Piglet Birth Weight (Day0) [kg]

M2 (Main Effect Model):

Random effects:

Group	n	Parameter	Variance	Std. Dev.
Piglet:Sow	69	(Intercept)<br>Time	0.0000<br>0.0004	0.0000<br>0.0199
Sow	24	(Intercept)<br>Time	1.2663<br>0.0224	1.1253<br>0.1497
Residual			1.7450	1.3210

Fixed effects:

Parameter	Estimate β	95% CI	Std. Error	P value
(Intercept)	0.4530	[-0.8990, 1.8026]	0.6774	0.5055
Time	-0.1707	[-0.2523, -0.0892]	0.0417	**<0.001**
Day0	-0.4484	[-1.3304, 0.4313]	0.4373	0.3088

M3 (Interaction Model):

Random effects:

Group	n	Parameter	Variance	Std. Dev.
Piglet:Sow	69	(Intercept)<br>Time	0.0000<br>0.0004	0.0000<br>0.0188
Sow	24	(Intercept)<br>Time	1.3044<br>0.0235	1.1421<br>0.1532
Residual			1.7508	1.3232

Fixed effects:

Parameter	Estimate β	95% CI	Std. Error	P value
(Intercept)	0.5982	[-2.1607, 3.3430]	1.3812	0.666
Time	-0.1943	[-0.5888, 0.2009]	0.1973	0.328
Day0	-0.5528	[-2.4851, 1.3911]	0.9700	0.570
Time:Day0	0.0170	[-0.2606, 0.2935]	0.1387	0.903

Model Comparison:

Model	npar	Marginal R^2	Conditional R^2	Log-Likelihood	P value
M1	9	0.1250	0.2984	-367.95	M2 vs M1: 0.3011
M2	10	0.1294	0.3053	-367.42	M3 vs M1: 0.5837
M3	11	0.1288	0.3072	-367.41	M3 vs M2: 0.9306

3.4 Piglet Weight after 24 Hours (Day1) [kg]

M2 (Main Effect Model):

Random effects:

Group	n	Parameter	Variance	Std. Dev.
Piglet:Sow	69	(Intercept)<br>Time	0.0001<br>0.0003	0.0080<br>0.0183
Sow	24	(Intercept)<br>Time	1.2510<br>0.0224	1.1184<br>0.1497
Residual			1.7430	1.3203

Fixed effects:

Parameter	Estimate β	95% CI	Std. Error	P value
(Intercept)	0.5686	[-0.7479, 1.8781]	0.6562	0.3885
Time	-0.1707	[-0.2523, -0.0885]	0.0417	**<0.001**
Day1	-0.4987	[-1.2941, 0.3011]	0.3945	0.2101

M3 (Interaction Model):

Random effects:

Group	n	Parameter	Variance	Std. Dev.
Piglet:Sow	69	(Intercept)<br>Time	0.0001<br>0.0003	0.0080<br>0.0177
Sow	24	(Intercept)<br>Time	1.2952<br>0.0236	1.1381<br>0.1536
Residual			1.7469	1.3217

Fixed effects:

Parameter	Estimate β	95% CI	Std. Error	P value
(Intercept)	0.8781	[-1.7502, 3.5288]	1.3235	0.509
Time	-0.2207	[-0.5988, 0.1560]	0.1898	0.248
Day1	-0.7075	[-2.4435, 1.0193]	0.8700	0.418
Time:Day1	0.0337	[-0.2155, 0.2835]	0.1249	0.788

Model Comparison:

Model	npar	Marginal R^2	Conditional R^2	Log-Likelihood	P value
M1	9	0.1250	0.2984	-367.95	M2 vs M1: 0.2022
M2	10	0.1319	0.3059	-367.14	M3 vs M1: 0.4299
M3	11	0.1315	0.3089	-367.11	M3 vs M2: 0.8038

3.5 Piglet Weight in 2nd to 10th Postnatal Week (Weight) [kg]

M2 (Main Effect Model):

Random effects:

Group	n	Parameter	Variance	Std. Dev.
Piglet:Sow	70	(Intercept)<br>Time	0.0004<br>0.0021	0.0203<br>0.0463
Sow	24	(Intercept)<br>Time	1.2590<br>0.0238	1.1221<br>0.1542
Residual			1.7001	1.3039

Fixed effects:

Parameter	Estimate β	95% CI	Std. Error	P value
(Intercept)	-0.2684	[-0.8647, 0.3288]	0.3035	0.385
Time	-0.0780	[-0.2379, 0.0802]	0.0803	0.333
Weight	-0.0383	[-0.0923, 0.0164]	0.0273	0.162

M3 (Interaction Model):

Random effects:

Group	n	Parameter	Variance	Std. Dev.
Piglet:Sow	70	(Intercept)<br>Time	0.0006<br>0.0016	0.0246<br>0.0405
Sow	24	(Intercept)<br>Time	1.3033<br>0.0238	1.1416<br>0.1543
Residual			1.6647	1.2902

Fixed effects:

Parameter	Estimate β	95% CI	Std. Error	P value
(Intercept)	-1.3471	[-2.4447, -0.2508]	0.5578	**0.0170**
Time	-0.0782	[-0.2343, 0.0791]	0.0794	0.3264
Weight	0.2362	[-0.0054, 0.4782]	0.1214	0.0535
Time:Weight	-0.0234	[-0.0434, -0.0033]	0.0101	**0.0218**

Model Comparison:

Model	npar	Marginal R^2	Conditional R^2	Log-Likelihood	P value
M1	9	0.1263	0.3148	-375.63	M2 vs M1: 0.1796
M2	10	0.1323	0.3417	-374.73	M3 vs M1: **0.0282**
M3	11	0.1494	0.3560	-372.06	M3 vs M2: **0.0209**

3.6 Colostrum Intake During the First 24 Hours (CI24) [kg]

M2 (Main Effect Model):

Random effects:

Group	n	Parameter	Variance	Std. Dev.
Piglet:Sow	69	(Intercept)<br>Time	0.0000<br>0.0000	0.0016<br>0.0063
Sow	24	(Intercept)<br>Time	1.1940<br>0.0226	1.0925<br>0.1503
Residual			1.7480	1.3221

Fixed effects:

Parameter	Estimate β	95% CI	Std. Error	P value
(Intercept)	0.3943	[-0.6618, 1.4384]	0.5296	0.4584
Time	-0.1707	[-0.2517, -0.0884]	0.0417	**<0.001**
CI24	-1.2587	[-3.2141, 0.7074]	0.9807	0.2027

M3 (Interaction Model):

Random effects:

Group	n	Parameter	Variance	Std. Dev.
Piglet:Sow	69	(Intercept)<br>Time	0.0000<br>0.0000	0.0034<br>0.0067
Sow	24	(Intercept)<br>Time	1.1910<br>0.0225	1.0915<br>0.1501
Residual			1.7560	1.3250

Fixed effects:

Parameter	Estimate β	95% CI	Std. Error	P value
(Intercept)	0.8583	[-1.1285, 2.8340]	0.9959	0.3906
Time	-0.2465	[-0.5329, 0.0392]	0.1438	0.0892
CI24	-2.2931	[-6.5067, 1.9241]	2.1203	0.2816
Time:CI24	0.1690	[-0.4399, 0.7829]	0.3068	0.5827

Model Comparison:

Model	npar	Marginal R^2	Conditional R^2	Log-Likelihood	P value
M1	9	0.1250	0.2984	-367.95	M2 vs M1: 0.1982
M2	10	0.1317	0.3034	-367.12	M3 vs M1: 0.3726
M3	11	0.1326	0.3030	-366.96	M3 vs M2: 0.5725

3.7 IgG Colostrum [log ELISA Units]

M2 (Main Effect Model):

Random effects:

Group	n	Parameter	Variance	Std. Dev.
Piglet:Sow	70	(Intercept)<br>Time	0.0000<br>0.0010	0.0025<br>0.0316
Sow	24	(Intercept)<br>Time	1.1600<br>0.0223	1.0771<br>0.1492
Residual			1.7540	1.3243

Fixed effects:

Parameter	Estimate β	95% CI	Std. Error	P value
(Intercept)	-1.1476	[-2.7694, 0.4655]	0.8055	0.1662
Time	-0.1737	[-0.2554, -0.0921]	0.0416	**<0.001**
IgG Colostrum	0.3358	[-0.1892, 0.8590]	0.2607	0.2119

M3 (Interaction Model):

Random effects:

Group	n	Parameter	Variance	Std. Dev.
Piglet:Sow	70	(Intercept)<br>Time	0.0000<br>0.0010	0.0000<br>0.0319
Sow	24	(Intercept)<br>Time	1.2243<br>0.0239	1.1065<br>0.1545
Residual			1.7537	1.3243

Fixed effects:

Parameter	Estimate β	95% CI	Std. Error	P value
(Intercept)	-1.7519	[-5.2065, 1.6868]	1.7613	0.331
Time	-0.0778	[-0.5721, 0.4158]	0.2519	0.760
IgG Colostrum	0.5457	[-0.6313, 1.7252]	0.6029	0.375
Time:IgG Colostrum	-0.0333	[-0.2025, 0.1357]	0.0862	0.703

Model Comparison:

Model	npar	Marginal R^2	Conditional R^2	Log-Likelihood	P value
M1	9	0.1263	0.3148	-375.63	M2 vs M1: 0.1887
M2	10	0.1363	0.3187	-374.76	M3 vs M1: 0.3879
M3	11	0.1370	0.3246	-374.68	M3 vs M2: 0.6835

3.8 IgG Serum [log ELISA Units]

M2 (Main Effect Model):

Random effects:

Group	n	Parameter	Variance	Std. Dev.
Piglet:Sow	70	(Intercept)<br>Time	0.0000<br>0.0025	0.0000<br>0.0496
Sow	24	(Intercept)<br>Time	1.7078<br>0.0271	1.3068<br>0.1645
Residual			1.5893	1.2607

Fixed effects:

Parameter	Estimate β	95% CI	Std. Error	P value
(Intercept)	1.1136	[0.0501, 2.1808]	0.5356	**0.0406**
Time	-0.2418	[-0.3375, -0.1453]	0.0488	**<0.001**
IgG Serum	-0.9572	[-1.5942, -0.3239]	0.3166	**0.0029**

M3 (Interaction Model):

Random effects:

Group	n	Parameter	Variance	Std. Dev.
Piglet:Sow	70	(Intercept)<br>Time	0.0000<br>0.0025	0.0000<br>0.0502
Sow	24	(Intercept)<br>Time	1.9091<br>0.0306	1.3817<br>0.1748
Residual			1.5709	1.2533

Fixed effects:

Parameter	Estimate β	95% CI	Std. Error	P value
(Intercept)	1.5713	[0.1587, 2.9918]	0.7083	**0.0289**
Time	-0.3171	[-0.5032, -0.1316]	0.0929	**<0.001**
IgG Serum	-1.3718	[-2.4229, -0.3219]	0.5240	**0.0098**
Time:IgG Serum	0.0788	[-0.0890, 0.2471]	0.0836	0.3469

Model Comparison:

Model	npar	Marginal R^2	Conditional R^2	Log-Likelihood	P value
M1	9	0.1263	0.3148	-375.63	M2 vs M1: **0.0049**
M2	10	0.1623	0.4064	-371.66	M3 vs M1: **0.0131**
M3	11	0.1660	0.4241	-371.29	M3 vs M2: 0.3874

3.9 IgM Serum [log ELISA Units]

M2 (Main Effect Model):

Random effects:

Group	n	Parameter	Variance	Std. Dev.
Piglet:Sow	70	(Intercept)<br>Time	0.0000<br>0.0015	0.0012<br>0.0388
Sow	24	(Intercept)<br>Time	1.1630<br>0.0225	1.0786<br>0.1501
Residual			1.7400	1.3192

Fixed effects:

Parameter	Estimate β	95% CI	Std. Error	P value
(Intercept)	-0.2144	[-0.7886, 0.3564]	0.2931	0.4718
Time	-0.2268	[-0.3528, -0.1008]	0.0642	**<0.001**
IgM Serum	0.1108	[-0.0907, 0.3127]	0.1016	0.2771

M3 (Interaction Model):

Random effects:

Group	n	Parameter	Variance	Std. Dev.
Piglet:Sow	70	(Intercept)<br>Time	0.0015<br>0.0005	0.0393<br>0.0232
Sow	24	(Intercept)<br>Time	1.0578<br>0.0191	1.0285<br>0.1383
Residual			1.6899	1.3000

Fixed effects:

Parameter	Estimate β	95% CI	Std. Error	P value
(Intercept)	-1.0798	[-1.8983, -0.2695]	0.4109	**0.0102**
Time	0.1404	[-0.1391, 0.4194]	0.1394	0.3161
IgM Serum	0.3313	[0.0797, 0.5847]	0.1271	**0.0102**
Time:IgM Serum	-0.0842	[-0.1420, -0.0264]	0.0289	**0.0041**

Model Comparison:

Model	npar	Marginal R^2	Conditional R^2	Log-Likelihood	P value
M1	9	0.1263	0.3148	-375.63	M2 vs M1: 0.2805
M2	10	0.1308	0.3214	-375.04	M3 vs M1: **0.0099**
M3	11	0.1612	0.3460	-371.01	M3 vs M2: **0.0045**
